# On the road to vision zero: How unit-dose dispensing systems and health-IT are transforming clinical practices

**DOI:** 10.1371/journal.pdig.0001023

**Published:** 2025-10-17

**Authors:** Saskia Herrmann, Natalie Bräuer, Tobias Zimmermann, Thomas Steiner, Dominic Fenske, Jana Gerstmeier

**Affiliations:** 1 Clinical Pharmacy, Helios Kliniken GmbH, Berlin, Germany; 2 Department of Pharmaceutical/Medicinal Chemistry, Institute of Pharmacy, Friedrich Schiller University Jena, Jena, Thuringia, Germany; 3 Clinical Pharmacy, Helios Klinikum Erfurt, Erfurt, Thuringia, Germany; 4 Department of Urology, Helios Klinikum Erfurt, Erfurt, Thuringia, Germany; 5 Medicine, HMU Health and Medical University Erfurt, Erfurt, Thuringia, Germany; University of Reading Reading School of Pharmacy, UNITED KINGDOM OF GREAT BRITAIN AND NORTHERN IRELAND

## Abstract

The study investigates real-world prescribing patterns and validation workflows linked to the implementation of a Unit-Dose Dispensing System (UDDS) within a digital medication management framework. The overall goal is to enhance medication safety, minimize errors, and improve clinical efficiency and workflow processes. Retrospective analysis of prescription data from the Electronic Medication System (EMS) in 2023 at a large tertiary care teaching hospital focused on physicians` prescribing patterns, drug compatibility with UDDS, and challenges faced by pharmacists during validation. Interactive dashboards provided real-time insights into prescription types, volumes, timing, and pharmacist validation rates. Of the 4.7 million doses prescribed in 2023, 64% were UDDS-compatible, highlighting its strong potential to streamline workflows and reduce nursing workload on the wards. Dashboard analysis revealed a clear alignment between peak prescribing times and UDDS production schedules, indicating effective synchronization between clinical and logistical workflows. Notably, an average of 631.6 blister-packable doses per day remained unvalidated by clinical pharmacists due to contraindications, dosage discrepancies, or duplicate prescriptions, emphasizing the need for enhanced health-IT support to address these gaps. UDDS combined with interactive dashboards enables targeted filtering and rapid identification of trends and gaps in pharmacotherapy. Integrating UDDS into a digital medication management framework offers significant potential to improve patient safety and operational efficiency. Key challenges in implementing UDDS into routine clinical practice were identified. Adhering to prescription submission cut-off times is essential to ensure UDDS effectiveness. Tailoring UDDS workflows and interactive dashboards to department-specific needs can further improve medication safety, strengthen pharmacists’ oversight, and support the long-term sustainability of safe and efficient medication practices.

## Author summary

Unit-Dose Dispensing Systems (UDDS) are transforming the hospital landscape. Innovative health-IT tools, such as interactive dashboards, enable smarter and safer medication management, that are tailored to the needs of each clinical department. While the safety benefits of UDDS are well-established, its effects on efficiency, usability and staffing remain underexplored. Our study shows that 64% of all prescriptions can be managed via UDDS, significantly easing nursing workloads on the wards. Advanced dashboards deliver real-time insights into physicians` prescribing patterns and timing, enhancing the clinical pharmacists’ workflows and supporting efficient UDDS production. Our findings underscore the importance of aligning pharmacy processes with ward routines to maximize the productivity of UDDS in a digital hospital setting.

## Introduction

Medication errors are a persistent challenge in healthcare, causing preventable harm and adverse outcomes. To tackle this, the WHO launched the “Medication Without Harm” initiative to enhance global medication safety [1]. The Vision Zero framework, originally developed for road safety [2], promotes a zero-tolerance approach to preventable harm - a principle we aim to apply to medication safety in clinical settings. A Vision Zero initiative for oncology already exists, highlighting the growing recognition of this approach in healthcare [3]. Vision Zero in medication safety could not only improve care but also cut overall costs.

Unit-Dose Dispensing Systems (UDDS) have emerged as a key strategy in reducing medication errors by enabling precise, patient-specific packaging of medications [4,5]. In Germany, the adoption of UDDS is particularly driven by the Hospital Future Act (“Krankenhauszukunftsgesetz”), a nationwide initiative that provides government funding to modernize and digitize the healthcare infrastructure [6,7]. The implementation of UDDS improves clinical workflows in alignment with the Closed Loop Medication Management (CLMM) model, that targets error-prone stages such as prescribing, administration, monitoring, and documentation. Key components of the CLMM include Electronic Medication Systems (EMS), pharmacists-led validation, automated drug dispensing, and digital documentation. The CLMM seeks to close the safety loop in medication delivery by streamlining workflows to minimize human errors and enhance patient outcome [8–11]. Within the CLMM framework, UDDS resembles the gold standard in robotic-assisted drug dispensing, along with automated dispensing cabinets [4,12,13]. Thus in 2020, UDDS was implemented at the tertiary care hospital in Erfurt (Helios Hospital Group, HK-EF), guided by the WHO’s “Medication Without Harm” initiative and the Hospital Future Act. Since then, patient-specific medication is provided for 1,125 beds in HK-EF [9]. The hospital strategically adopted UDDS to blister pack solid oral medications into combi-doses. These contain all units of the same drug (e.g., multiple ibuprofen tablets) prescribed for a specific administration time. This approach ensures that patients receive all prescribed medications in a convenient and accurate format [14,15]. As previously described, UDDS is well-received by nursing staff, significantly reduces drug stocks on the wards, cuts medication dispensing time by 50%, and annually relieves up to 11.7 full-time equivalent (FTE) nursing staff per 1,000 beds. However, operating the UDDS department in the hospital pharmacy requires additional FTEs here [9]. Consequently, the transformation requires adequately trained pharmacy staff to adapt to the changing demands [16] as UDDS implementation introduces significant changes to the roles, daily routines, and workflows of both clinical and pharmacy personnel [4,5,8,9,14,17].

Successful UDDS implementation depends on overcoming several key challenges. First, not all medications are suitable for blister packaging. The *Data Collection for the Assessment of Oralia* (DaBO) provides essential guidance by classifying pharmaceutical forms based on properties such as moisture, light and oxygen sensitivity [18]. Yet, we lack overall real-world data on medication compatibility with UDDS. Second, ward workflows must be adapted to ensure timely medication delivery. This involves aligning the timing of medical rounds and EMS prescription entries with UDDS production schedules. Typically, unit-dose blister packs are prepared on the same day a prescription is entered into the EMS, allowing patients to receive their medications the following day [5,9,19,20]. Without proper integration into ward routines, delays and disruptions in medication delivery can occur [21–23]. Third, certain medications such as narcotics and T-prescriptions (e.g., thalidomide) are excluded from UDDS due to strict regulatory requirements [24–26]. These drugs require manual handling, which interrupts automation, complicates workflows, and increases the potential for errors [8,27,28]. Finally, maintaining a safe and efficient UDDS process requires continuous validation of the prescriptions by pharmacists, including weekends. In the UDDS context, pharmacist validation refers to the systematic review and verification of medical prescriptions by a pharmacist to ensure appropriateness, accuracy, and safety prior to medication dispensing, with particular attention to dosing, potential drug interactions as well as contraindications [8,22].

Together, four key factors are essential for successfully integrating UDDS into both existing and emerging clinical workflows. These include: (I) the feasibility of blister-packing high volumes of medications, (II) alignment of delivery schedules with ward-specific routines and medical rounds, (III) the potential need for weekend blistering services, and (IV) continuous validation of patient-specific medications throughout the week. Interactive dashboards integrated into the UDDS workflow serve a dual purpose here. We attempt to show that these dashboards provide real-time insights into prescribing patterns, flag high-risk drug classes, and contibute to align pharmacy services with clinical routines [28,29].

Despite its growing adoption, there is limited published evidence of real-world operational data, implementation challenges, and the broader impact of UDDS on economic and safety outcomes. This study aims to address this gap by leveraging interactive dashboards to analyze real-world patterns in prescribing and pharmacist validation performance. The primary goal is to identify drug usage patterns most compatible with UDDS, enabling more efficient, targeted medication delivery and optimized pharmaceutical management. Finally, the study provides actionable insights into the effective integration of UDDS within a fully digital hospital environment.

## Materials and methods

### Data source and study design

Prescription data from 2023 were retrospectively analyzed using ID EFIX PHARMA (version 12.0.0.600) [30]. ID EFIX PHARMA is a tool developed for retrospective database analysis, enabling a variety of evaluations that can contribute to improving patient care. Its modular design allows users to create customized dashboards with advanced filter settings, enabling precise and flexible analysis of medical data. Originally developed for billing patient cases, ID EFIX PHARMA has been adapted to seamlessly interface with the Hospital Information System (HIS) and the Electronic Medication System (EMS). This integration enables automated data transfer and real-time access to medication and patient-related information, supporting more efficient clinical workflows and data-driven decision-making. In the EMS, each drug is prescribed with defined parameters such as active ingredients, dosage, form, and route of administration. These data are transferred to ID EFIX PHARMA, enabling detailed analysis of prescriptions using customizable filters and dashboards (S1 Fig). The software supports evaluations based on anonymized patient information (e.g., age, gender), medication details, dosage intervals, and alerts. All patient identifiers (e.g., case number, date of birth) and personnel-related data (e.g., physician signatures) were excluded to ensure full compliance with data protection regulations.

### Dashboard structure, inclusion criteria and filter logic

All dashboards in this study follow a standardized structure and analyze three main categories “prescriptions”, “prescribed doses” and “documented doses” based on filter settings I-IV (see S1 Table and S2 Fig). The corresponding inclusion criteria for each filter, defined in ID EFIX PHARMA, are presented in S2 Fig. The analysis was limited to 2023 inpatient admissions on wards supplied by UDDS. Accordingly, intensive care units and pediatric wards were excluded, as they are not part of the UDDS supply at HK-EF. A drug’s eligibility for blister packaging (“blisterability”) is determined by its pharmaceutical form and prescription type, both are filtered in the respective dashboards. The validation status indicates whether the clinical pharmacist has reviewed and approved the prescribed medication.

### Prescriptions sorted by pharmaceutical form

The filter setting I (“prescribed drugs”) was used in combination with the documentation status, which allowed for the analysis of parameters such as pharmaceutical form, prescription type, and prescribed dose. Drugs were grouped into five main pharmaceutical form categories: solid, liquid, semi-solid, inhalant, and other (see S2 Table). Within the solid group, a further classification was applied based on two criteria: blisterability and prescription type (see S3 Fig). This resulted in two subcategories of solid drugs: solid, blisterable drugs (= compatible with UDDS) and solid, non-blisterable drugs (not compatible with UDDS, w/o). Results were visualized as a dot plot using GraphPad Prism 10 (v10.1.2).

### Prescription timing and volume analysis

This analysis assessed both the amount and timing of prescriptions as well as corresponding prescribed doses. A prescription represents a medication order (e.g., ibuprofen 400 mg, three times daily), while prescribed doses represent the total number of individual doses resulting from the prescription (e.g., 3 doses per day × 5 days = 15 prescribed doses). Documented doses refer to prescribed doses that were confirmed and recorded as administered to the patient.

Filter settings I–IV (see S2 Fig) were applied to evaluate the number of prescriptions, prescribed doses, documented doses, departments, and number of patients. Based on these data, the documentation rate was calculated as the proportion of documented doses relative to prescribed doses (see S1 Table, S4A Fig). Filter settings I, III, and IV were used to calculate the average daily totals of prescribed, blisterable, and unvalidated blisterable doses. These values were used to reflect daily averages for 2023 (S4B Fig). For the temporal analysis, filter setting II was applied to examine prescription timing patterns by each ward (S4C Fig). Each prescription timestamp was assigned to a time interval (e.g., a prescription at 04:53:28 was mapped to the 03:00–05:00 interval), and then grouped by weekday. The time intervals used were: 00:00–03:00, 03:00–05:00, 05:00–07:00, 07:00–09:00, 09:00–11:00, 11:00–13:00, 13:00–15:00, 15:00–17:00, 17:00–19:00, 19:00–21:00, and 21:00–00:00. Results were visualized as heatmap using GraphPad Prism 10 (v10.1.2).

### ATC based evaluation of non-validated blisterable drugs

Each drug prescribed in the EMS is linked to a corresponding Anatomical Therapeutic Chemical (ATC) classification, analyzed using ID EFIX PHARMA. The unique assignment of ATC codes to pharmaceutical agents allows a standardized evaluation independent of specific manufacturers [31]. Filter settings I, II, and IV were applied here to assess the number of prescriptions per 2^nd^ level ATC code (S5 Fig). Data were visualized as radar plot and heat map, created with Microsoft Excel 2016 (v16.0.5448.1000).

### Ethical considerations

The study adhered to institutional and ethical guidelines for research involving operational data. No patient-specific data were collected, ensuring compliance with data protection standards.

### Statistical analysis

The statistical analysis was performed using GraphPad Prism 10 (version 10.1.2). The normality of the data distribution was assessed using the Kolmogorov-Smirnov test for sample sizes greater than 50, and the Shapiro-Wilk test for sample sizes of 50 or fewer [32]. For data not conforming to normal distribution, statistical analyses were conducted using the Wilcoxon test as indicated. The results are presented as mean ± standard error of the mean (SEM) or sum depending on data presentation. A *p*-value of < 0.05 is considered statistically significant.

## Results

### Insights into prescribing patterns and pharmaceutical suitability

The UDDS workflow at HK-EF includes drug suitability assessment, prescription validation by clinical pharmacists, blister packaging, and final inspection, followed by delivery to wards. Non-validated or adjusted prescriptions require physician consultation and are manually dispensed by nursing staff, as illustrated in S6 Fig.

In 2023 at HK-EF, a total of 416,361 prescriptions were issued for inpatients, corresponding to 4,706,731 prescribed doses for 42,070 patients. On average, each patient received 9.9 prescriptions and 111.9 prescribed doses over the course of their hospital stay. A detailed data analysis for each hospital department is shown in Table 1. Column six presents the documentation rate. The documentation rate is shown as heat map, with red indicating low documentation rates and blue representing high documentation rates. It is evident that the documentation rate for blister-packaged and validated drugs is higher than for total prescribed drugs (Table 1, filter setting III vs. I). Department I was subdivided according to its three distinct cut-off times for EMS entries, which determine the UDDS validation and production schedules. Section Ia, with a cut-off time of 11:00 o’clock, primarily includes surgical wards. Section Ib, with a cut-off time of 13:00 o’clock, covers internal medicine. Section Ic, with a cut-off time of 14:00 o’clock, is composed predominantely of internal medicine wards (1 and S3 Tables).

**Table 1:** Descriptive data of prescriptions, prescribed and documented doses of HK-EF in 2023 by department.

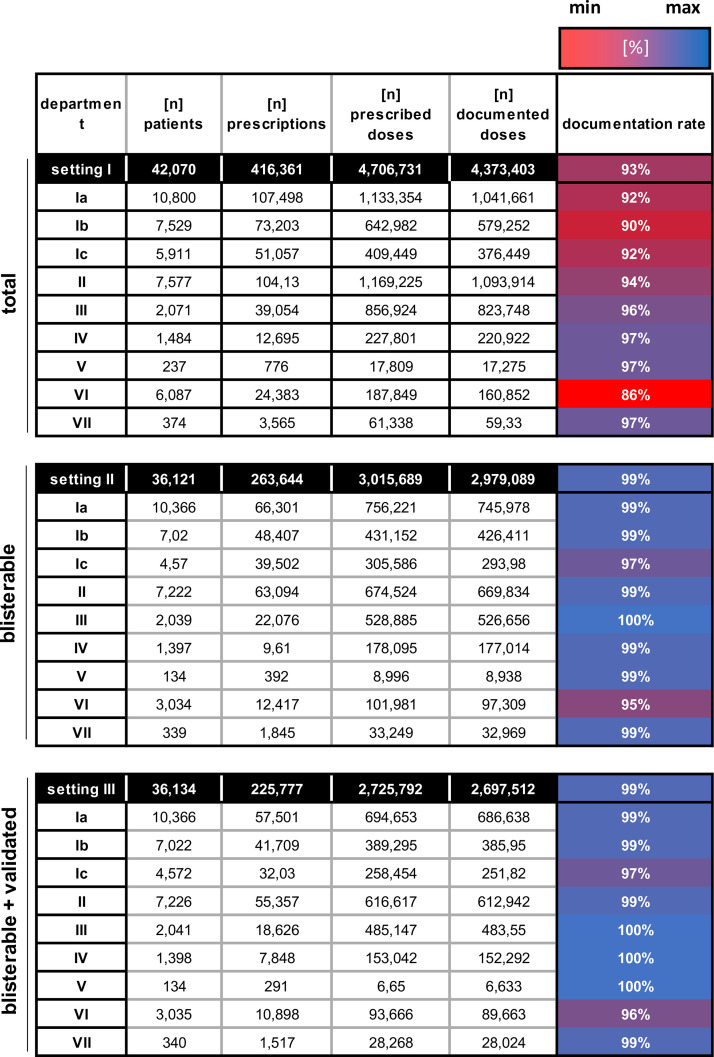							

Filter settings I-III, representing “total” (I), “blisterable” (II), and “validated + blisterable” (III) drugs. Filter settings are subdivided by departments I-VII. Documentation rate is shown as heatmap in %, representing the proportion of documented relative to prescribed doses (low = red; high = blue).

**Fig 1:**
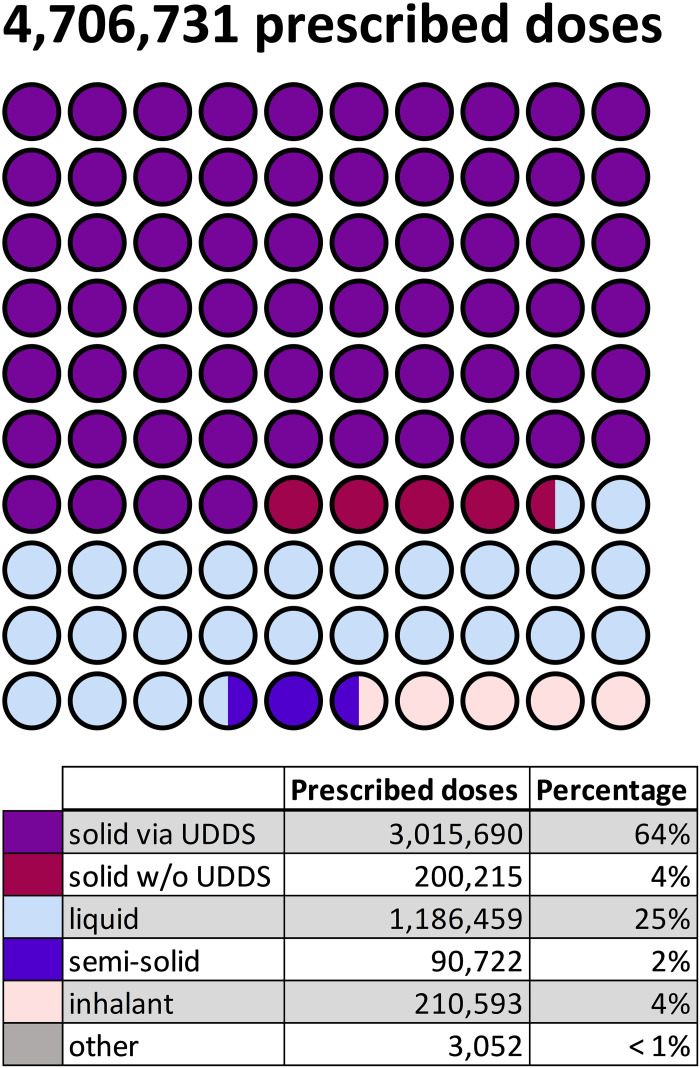
Distribution of prescribed doses according to pharmaceutical form at HK-EF in 2023. Solid pharmaceutical forms administered via UDDS are represented in purple, non-bliserable solid forms in red, liquid forms in light blue, semi-solid forms in dark blue, inhalants in apricot and other pharmaceutical forms in grey. The frequency of prescriptions for each pharmaceutical form is illustrated using a dot plot and shown as absolute numbers in the table below. UDDS = Unit-Dose Dispensing System, w/o = without.

Of the total 4,706,731 prescribed doses (Table 1), 68% consisted of solid pharmaceutical forms (Fig 1). The remaining 32% included liquid forms (= 25%; such as injection solutions, suspensions, and infusions), semi-solid forms (= 2%; including creams and ointments), inhalants (= 4%; such as metered dose and powder inhalers) and other forms (< 1%; e.g., inserts and patches). Among the solid pharmaceutical forms, 94%, accounting for 3,015,690 prescribed doses, were considered suitable for distribution via UDDS. The 6% of solid, non-blisterable drugs include, e.g., effervescent or chewable tablets with high friability, as well as narcotic medications that cannot be blister-packed due to legal restrictions (Narcotic Drug Act) [26]. Thus, 64% of the total prescribed doses were suitable for UDDS (purple dots, Fig 1).

**Fig 2:**
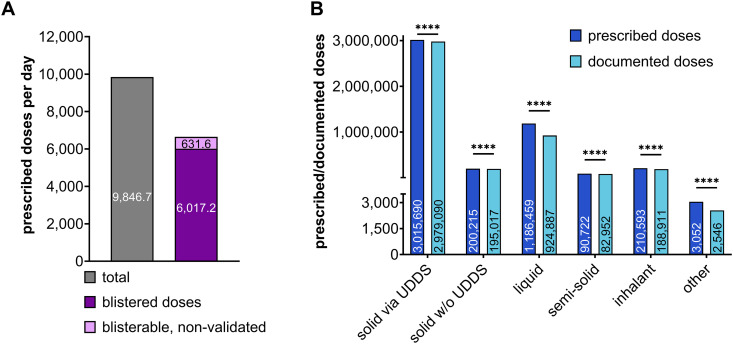
2023 Prescription and documentation overview of UDDS-supplied wards. All data are shown as mean throughout the year 2023. A: Average number of all prescribed doses per day (grey bar). The purple bar indicates the average blisterable and validated prescribed doses per day, and the light purple bar shows the non-validated doses. B: Total number of prescribed (dark blue) and documented (light blue) doses in 2023, shown as cumulative sums across the various pharmaceutical forms. Data were not normally distributed. The Wilcoxon rank-sum test was used for further statistical analyses using original, unprocessed datasets (****p < 0.0001; refer to S1 Data).

The average daily number of prescribed doses at HK-EF in 2023 was 9,846.7 ± 323.6 (mean ± SEM), as shown in Fig 2A (grey bar). Of these, 61% (purple bar) were successfully blister-packed and validated each day. However, an average of 631.6 doses per day (light purple bar), although eligible for blister packing, remained non-validated by the clinical pharmacist. Here, pharmacists intentionally withhold validation to prevent the potential administration of inappropriate medication to the patient.

Documentation rates, illustrated as heatmap in Table 1, represent the proportion of blister-packable prescribed doses that were correctly documented. These rates provide essential insights into the effectiveness of medication management. Importantly, without documentation, the medication process is legally incomplete. As shown in Fig 2B, solid blisterable drugs dominate prescriptions, accounting for 3,015,690 doses, with a documentation rate of 99%. Non-blisterable solid pharmaceutical forms also demonstrate strong performance, with a documentation rate of 97%. Liquid (78%), semi-solid (91%), and inhalant formulations (90%) all demonstrate lower documentation rates, highlighting challenges in documentation particular for non-solid forms. “Others” display a gap as well, with only 83% of the 3,052 prescribed doses being documented. Despite the described differences, all pharmaceutical forms lack proper documentation and should be optimized in the future (Fig 2B) with an overall documentation rate of only 93% across all pharmaceutical forms (Table 1).

### Temporal prescription patterns across clinical departments

The number of prescribed doses eligible for UDDS varies significantly across different clinical departments I-VII (1 and S3 Tables), depending on several factors such as the number and size of the wards, the medical field, and the timing of ward rounds or prescription entry into the EMS. These factors play a crucial role in determining how effectively UDDS can be integrated into each department’s workflow.

**Fig 3:**
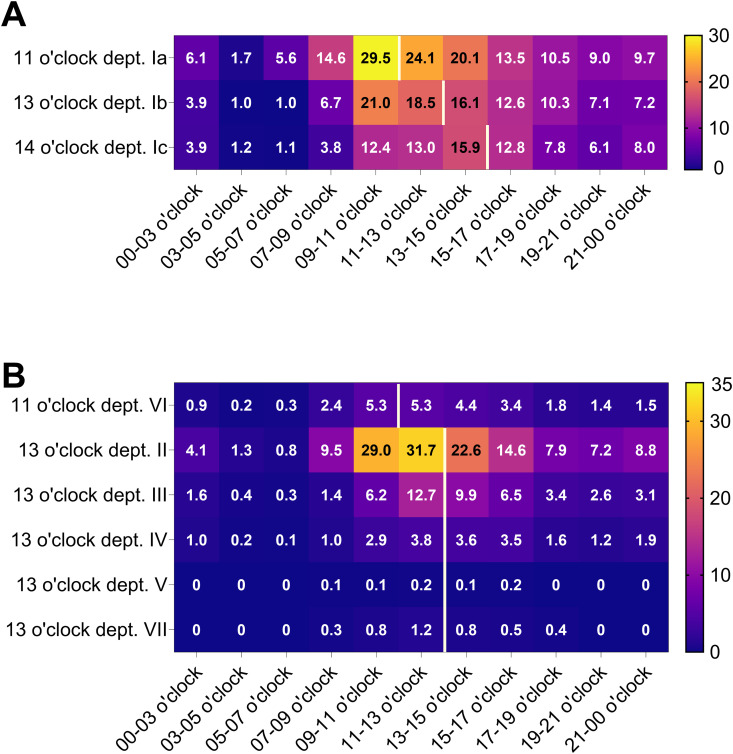
Time interval analyses of prescription entries across departments in 2023. Results are presented as average number of prescriptions per day during 2023 and shown as heatmap. Blue to dark purple cells indicate low prescription volumes, while yellow reflects high prescription numbers. The white line indicates respective UDDS cut-off times. Department (dept.) Ia-c is shown in A and dept. II-VII in B.

The results for HK-EF are displayed as heatmap in Fig 3, with blue and purple highlighting low numbers of prescription entries and yellow representing high prescription entries. For department Ia the highest volume of prescriptions are during 9:00–11:00 o´clock. During this period, an average of 29.5 prescriptions were entered into the EMS, highlighting the peak activity in medication orders for this department (Fig 3A). Department II exhibits the highest prescribing activity between 11:00 and 13:00 o’clock, with an average of 31.7 prescriptions per day (Fig 3B). In contrast, department V had the lowest level of prescribing activity with less than one prescription per day during the same time frame (Fig 3B). As expected, more prescriptions are issued on weekdays compared to weekends (S7 Fig).

### Spotlight on safety: Addressing non-validated prescriptions

**Fig 4:**
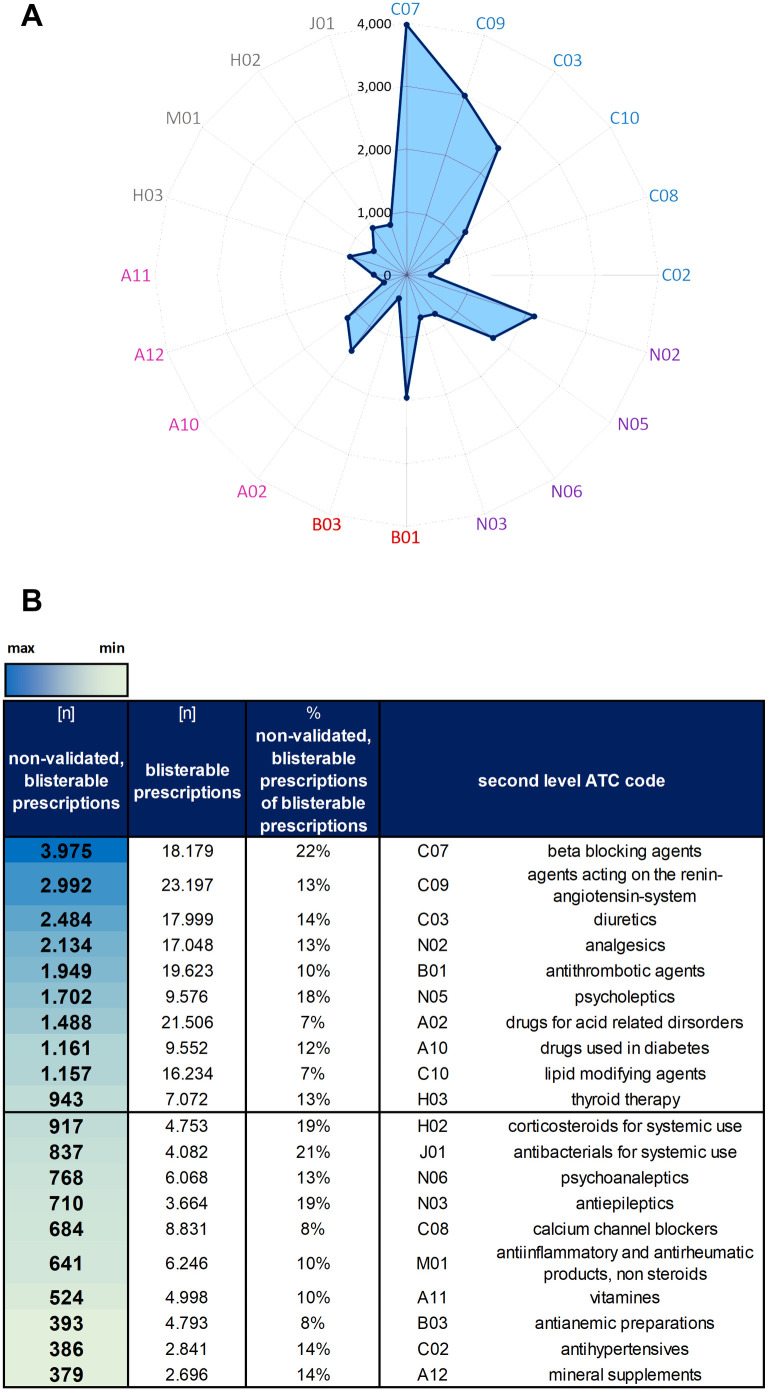
Non-validated blisterable prescriptions categorized by second-level ATC codes. A: The 20 most frequent 2^nd^ level ATC codes of non-validated blisterable prescriptions are arranged as radar plot. ATC codes are grouped and sorted in descending order. Prescriptions targeting the cardiovascular system are highlighted in blue, nervous system in purple, blood-related drugs in red, and those affecting the alimentary tract and metabolism in pink. All remaining ATC codes are shown in gray. B: Absolute numbers of non-validated blisterable prescriptions for each second level ATC code are ordered in the table including a heat map from high to lower levels. Blue indicates a high number of non-validated prescriptions, while lighter green shades indicate lower numbers. 1-10 are shown in the upper half, 11-20 in the lower half.

As illustrated in Fig 2A, not all prescriptions are validated by clincal pharmacists. Non-validated prescriptions have the potential to contain errors or issues that pose a threat to patient safety. Non-validated medications lead to workflow interruptions on the ward, making it important to identify which medications are most affected. On average, 631.6 (10%) prescribed doses remain non-validated per day (Fig 2A). Thus, we analyzed the 20 most prescribed drugs without pharmacist approval (Fig 4A). These findings were further compared to their proportion of blisterable prescriptions (Fig 4B). Notably, beta-blockers (ATC C07) constituted the largest category of non-validated medications. Despite ranking fourth in overall blisterable prescriptions (S4 Table), with a total of 18,179 prescriptions issued, a significant number of 3,975 prescriptions remained non-validated. This represents 22% of all blisterable beta-blocker prescriptions in 2023, underscoring a critical gap in the validation process (Fig 4 and S4 Table). In absolute numbers, medications targeting the blood pressure ranked second and third position among the non-validated prescriptions (Fig 4B), highlighting their sensitive role in therapeutic regimens. Although ATC group J01 with 837 non-validated prescriptions ranked position twelve in terms of absolute numbers, it resembles the second place when ranked by percentage of non-validated blisterable prescriptions, accounting for 21% (Fig 4). This highlights the importance of J01, as it includes essential drugs for treating bacterial infections. A similar pattern was seen for systemic corticosteroids (ATC H02), with 19% prescriptions remaining non-validated by the pharmacist.

## Discussion

Successful UDDS integration requires more than technical adjustments in the hospital pharmacy. It demands a strategy that addresses regulatory, financial, and logistical challenges. By leveraging advanced dashboards and optimizing staff workflows, hospitals can implement and maintain UDDS effectively, enhancing medication safety, operational efficiency, and overall care quality. However, comprehensive data on the successful implementation and long-term maintenance of UDDS remain limited. Thus, the primary goal of this study was to explore the practical benefits and challenges of UDDS integration in routine hospital operations. In particular, we sought to identify internal processes that may be influenced or require adjustments due to UDDS implementation. UDDS has already been shown to significantly reduce nursing staff workload [9,17,22]. Our current study focuses on physicians’ prescription patterns, pharmacist`s validation and workflow, and to which extend UDDS could automate the requested CLMM.

### UDDS automation potential

To begin with, 64% of all prescribed doses are suitable for delivery via UDDS, while 36% still require manual preparation by nursing staff (Fig 1). A total of 4,706,731 doses were prescribed in 2023 in HK-EF. Due to physical limitations (e.g., chewable or effervescent tablets) [18] and legal restrictions (Narcotic Drug Act [26]), 200,215 doses of solid medication (≙ 4%) cannot be blister-packed. A notable limitation of UDDS is the inability to package liquid formulations, which represent 25% of all prescribed doses (Fig 1), accounting for 1,186,459 doses per year. While alternative systems do exist that can manage liquid medications, these solutions come with their own advantages and limitations [33,34]. Implementing UDDS can be costly, requires significant initial investment in technology and extensive staff training. Despite the current limitations, technological advancements are evolving. For instance, a french start-up has developed a system specifically designed for liquid pharmaceutical forms [34]. Similarly, a swiss manufacturer offers blister-packaging technology that accommodates both solid oral medications and ampoules, expanding the scope of automated medication management [33]. Moreover, automated dispensing cabinets provide the potential for preparing most dosage forms on an individual basis for each patient [5]. But in contrast to UDDS, this process occurs on the wards, which does not reduce the workload for the nursing staff [9]. Therefore, UDDS is currently the preferred robotic-based dispensing of medications regarding patient safety.

### Documentation quality as key for financial reimbursement

High-quality documentation is essential for CLMM compliance and financial reimbursement, especially within the German DRG framework (diagnosis related group). Thus, maintaining clear, accurate, and up-to-date documentation is crucial. For all blisterable medications, a documentation rate of 99% was evident versus only 78% for liquids, indicating a critical need for improvement here (Fig 2B). By tailoring strategies for low-performing areas, hospitals can optimize their medication management systems and improve patient safety outcomes as well as financial reimbursement. Departmental variations also suggested that patient autonomy and nursing involvement significantly impact documentation quality. Department VI for example exhibited the lowest documentation rate, whereas department III recorded the highest (Table 1). Patients in department VI are generally in good health and manage their own care, with nursing support if needed. Their high level of personal responsibility likely contributes to the lower documentation rate by the nurses [12,35]. In contrast, patients of department III are not able to receive UDDS blister packs for the entire day. In this setting, the nursing staff is responsible for administering medications at each scheduled time. Consequently, each drug must be documented by the nurses. This likely explains the high documentation rate for blisterable medications here. However, further analysis is needed to ensure proper UDDS use and elevate compliance on every ward.

Next, the German healthcare system relies on the DRG system for hospital financing and patient care. As already mentioned, we found that UDDS plays a key role in documenting medication administration (Fig 2B). From an economic viewpoint, this is especially important for high-cost medications, such as innovative drugs or treatments that fall outside the standard hospital budget, e.g., supplementary reimbursement (“*Zusatzentgelt*”, ZE) or new treatment methods („*neue Untersuchungs- und Behandlungsmethoden“,* NUB; §6 *Krankenhausentgeltgesetz*). Given the importance of proper documentation for both clinical management and financial accountability, UDDS serves as a valuable tool to ensure complete reimbursement.

### Dashboard-driven prescription pattern analysis and validation insights

Prescription timing analysis revealed alignment with the cut-off times for UDDS production in most departments (Fig 3). However, in department Ia, a high number of prescriptions continue to be issued beyond the designated UDDS cut-off period (Fig 3A). Particularly on surgical wards, on-demand medications, e.g., for proper pain management are frequently prescribed postoperatively, and thus cannot be blister-packed in advance. Additionally, new patient admissions after 16 o’clock disrupt the UDDS process. As expected, weekend and weekday prescription patterns also varied with more prescriptions issued on weekdays (S7 Fig), offering valuable data to optimize staffing and production planning. S7 Fig is particularly of interest when considering the diverse interests of the stakeholders involved. For example, hospital management prioritizes minimizing overtime and shift work to reduce operational costs. At the same time, staff members value having weekends off. However, the length of inpatient treatment is driven by clinical needs rather than administrative preferences. This makes a careful and balanced evaluation essential to safeguard the well-being of all parties. In department I-IV for instance, more than 200 prescriptions per ward are issued on an average weekend day. Currently, all departments, except department V, receive blister-packed medications also on saturdays. If additional staff capacity is required, e.g., to supply an external hospital via UDDS, the 200-prescription threshold per ward may serve as a benchmark for determining production capacity.

Next, the study identified that many commonly prescribed drugs, especially cardiovascular (ATC code C07 and C09), antibacterial (ATC code J01), and psychotropic (ATC code N05 and N06) medication, frequently remained non-validated by the clinical pharmacists (Fig 4). Reasons include dose deviations, contraindications, and duplicate entries, often due to fragmented communication or incomplete lab data [36,37]. This underscores the importance of pharmacist oversight and the implementation of strong validation systems. Importantly, the 20 most non-validated drugs from Fig 4 correspond to the overall list of the 20 most prescribed and blisterable drugs (S4 Table), indicating that most non-validated prescriptions are also commonly prescribed drugs. In fact, the most non-validated ATC codes in our study align well with the ATC codes from a previous study on avoidable medication harm [29]. This consistent pattern suggests that the non-validated medications in our study may carry a higher risk of preventable harm, underscoring the need for comprehensive validation and careful dose management within these drug categories. Here, data-driven interactive dashboards are key for smart and on demand health-IT support.

In detail, cardiovascular agents account for the majority of non-validated prescriptions (Fig 4A). Here, 1,949 out of 19,623 blisterable antithrombotic prescriptions were non-validated. These drugs require precise dosing and monitoring [38], making a pharmacist validation vital. Next, the high rate of non-validated J01 prescriptions raises serious concerns, as it increases the risk of antimicrobial resistance and medication errors, such as incorrect dosing or inappropriate drug selection, thereby worsening an already critical global healthcare challenge. In fact, ensuring accurate prescribing practices is essential to prevent overuse or misuse of these medications. Since J01 drugs are often used to treat severe infections, any lack of validation can directly impact patient safety and treatment outcomes. Similarly, mental health (ATC code N05) and analgesic medications (ATC code N02) exhibited high validation gaps due to complexity and interaction risks. Here, psycholeptics (N05) stand out, with 1,702 non-validated prescriptions and analgesics rank fourth in terms of absolute numbers, with 2,134 non-validated prescriptions. This underscores the potential challenges in achieving accurate dosing and managing patient-specific contraindications in pain management. Note that all these medications often necessitate validation due to their potential for drug interactions and significant side effect risks (Fig 4). Together, the identification of vulnerable drug classes and validation gaps underscore the urgent need for enhanced pharmacist oversight, particularly in high-risk therapeutic areas like blood pressure management and hormone regulation (Fig 4). Our data strongly support the pharmacists’ precautionary decision to withhold validation of certain prescriptions to prevent potential patient harm. Interactive dashboards offer here smart and efficient support by providing real-time insights and enabling prompt decision-making. Identifying the most affected medication groups is key here to optimize the validation process, which will be content of a future approach. Interactive dashboards will play a key role in achieving high medication safety by providing real-time feedback that supports continuous workflow improvements and reinforces safety measures. Data from Fig 4 supports this proactive risk management.

### Study limitations and future directions

The study design presents certain limitations, notably the lack of data on prescriber experience and clinical context (e.g., routine vs. emergency prescriptions), and the omission of pharmacist validation timing (Fig 2). Implementing UDDS comes with considerable operational demands [39,40], including risks related to system failures and a poor digital infrastructure. Financial costs and practical challenges must be considered as well [41], particularly the need for workflow reconfiguration to accommodate new technologies and the recruitment of new personnel [17]. Thus, future work should focus on real-time monitoring of phamacist validation patterns, and the development of adaptive UDDS schedules based on these dynamic data.

When closely tailored to clinical needs, UDDS - supported by these dashboards - becomes a powerful tool that not only enhances the hospital economic performance but also supervises key therapeutic trends [1,42]. Aligning UDDS workflows with department-specific routines, as illustrated in Fig 3, was identified as a key factor in enhancing system efficiency and supporting the intended CLMM principles [11,14]. The analysis of prescription patterns and documentation rates further contributes to an optimized UDDS setup (Table 1). In the future, personalized, on-demand unit dose packaging through 3D printing holds promise for tailoring medication delivery to individual patient needs as well [43,44]. Finally, integrating barcode scanning technologies (e.g., Bar-Coded Medication Administration) represents the logical next step to enhance traceability and significantly reduce Medication Administration Errors (MAE) [41,45] to fully adapt a digital medication management framework.

## Conclusion

The study underscores both the advantages and challenges associated with implementing UDDS in established hospital workflows. While 64% of prescribed doses are compatible with automated blister-packaging, certain medication forms like liquids, semi-solids, and narcotics still require manual handling, highlighting areas for further optimization. The detailed analysis of second-level ATC codes for non-validated blisterable drugs identifies critical drug classes that demand focused attention of the pharmacist to minimize patient harm. In the future, hospitals should integrate health-IT dashboards for smart and rapid support to realize the full potential of UDDS. Our study highlights how these dashboards can streamline workflow adjustments, monitor prescription timing, enhance operational efficiency and most importantly support physicians and pharmacists in achiving a high standard of medication safety.

## Supporting information

S1 Data Original dataset for statistical analysis used in Fig 2B. The pharmaceutical form is shown according to prescription type with the number of prescribed and documented doses. The dosage form determines the respective main group of the pharmaceutical form, and the combination of the pharmaceutical form and the prescription type determines the blister packability.
(DOCX)

S1 Table Glossary of terms. (DOCX)

S2 Table Prescribed doses by subordinate pharmaceutical form. Solid forms eligible for unit-dose are ranked in descending order by absolute prescription frequency. Drugs with modified release of the active ingredient are categorised within the superior pharmaceutical form (e.g., enteric-resistant capsules are classified as capsules).
(DOCX)

S3 Table Wards per department. Total number of wards assigned to each hospital department at HK-EF. Each row corresponds to a specific department, and the adjacent column indicates the number of wards managed by that department. Dept. I is subdivided in Ia-c.
(DOCX)

S4 Table Top 25 prescribed ATC-Codes in HK-EF in 2023. The top 25 most frequently prescribed ATC codes at HK-EF in 2023 are presented in a heat map, ranked in descending order by absolute prescription frequency. The first row displays the absolute numbers of prescriptions, with blue indicating the highest and light green the lowest. The second row shows the corresponding 2^nd^ level ATC codes, while the third row provides their full descriptions.
(DOCX)

S1 Fig Schematic overview of data exchange. The hospital information system (HIS) transfers administrative patient data to the electronic medication system (EMS), which is used by physicians for drug prescribing. The EMS can generate clinical alerts, such as contraindication warnings or weight-adjusted dosing recommendations, but does not support prescription data analysis. ID EFIX PHARMA consolidates data from both the HIS and EMS, providing structured data fields that enables various types of evaluations based on defined criteria for dashboards.
(DOCX)

S2 Fig Graphical overview and tabular description of filter settings I - IV. Filter setting I includes all prescribed drugs without limitations. Filter setting II refines the selection to prescribed and blisterable drugs. Filter setting III further narrows the focus to prescribed, blisterable, and validated drugs. Filter setting IV identifies prescribed, blisterable, but non-validated drugs. w/o = without, special order = drugs not routinely stocked.
(DOCX)

S3 Fig Schematic overview of filter setting applied to pharmaceutical form. The flowchart illustrates the stepwise application of filter setting I in combination with prescription status to classify medications by pharmaceutical form. Drugs are first grouped into five categories: solid, liquid, semi-solid, inhalant, and other. Solid drugs are further divided based on blisterability and prescription type, resulting in classification as UDDS-compatible (solid UDDS) or not compatible (solid w/o UDDS).
(DOCX)

S4 Fig Flowcharts for the dashboard analysis of prescription timing and volume. (A) Overview of parameters and filter settings I–III used to extract descriptive prescription data. (B) Workflow to calculate average daily prescribed doses using filter settings I, III, and IV. (C) Approach to analyze prescription time distribution, including time clustering and weekday assignment. Steps performed in Microsoft Excel are highlighted in grey.
(DOCX)

S5 Fig Flowchart for 2^nd^ level ATC codes analysis. Filter settings I, II and IV were used to identify and compare the 20 most frequently prescribed 2^nd^ ATC codes (lightblue), across all prescriptions (grey), blisterable prescriptions (dark purple), and non-validated blisterable prescriptions (light purple).
(DOCX)

S6 Fig Workflow overview of UDDS at HK-EF. The supply of a ward with UDDS is contingent upon the determination of the feasibility of blister packing the medication. In the event that the items are not available in bulk, they must be deblistered. Concurrently, clinical pharmacists evaluate patients’ medications for potential discrepancies, including overdoses or underdoses, duplicate prescriptions, and interactions. All medications that have been validated are packaged individually for each patient using UDDS and delivered to the wards after a final visual inspection for correctness of the blister packed drug. Next, the blister packs are adminsistered to the patients by the nursing staff. Moreover, the nursing staff controls the delivered UDDS for correctness with the prescription entry in the EMS. If the medication is not produced due to pharmaceutical concerns, the nursing staff consults the attending physicians. The attending physician determines whether the medication is distributed from the ward stock or whether a dose adjustment or discontinuation is necessary according to the pharmacist´s concerns.
(DOCX)

S7 Fig Average prescribed doses per weekkday versus weekend. Bar chart illustrates the mean number of blisterable prescribed doses on working days (black) and weekends (pink) for each clinical department in 2023. dept. = department.
(DOCX)
