# Supplementary material for: On the road to vision zero: How unit-dose dispensing systems and health-IT are transforming clinical practices
Source: PLOS Digit Health. 2025 Oct 17;4(10):e0001023. doi: 10.1371/journal.pdig.0001023 (PMC12533864; doi:10.1371/journal.pdig.0001023)
Supplement: S1 Data — The pharmaceutical form is shown according to prescription type with the number of prescribed and documented doses. The dosage form determines the respective main group of the pharmaceutical form, and the combination of the pharmaceutical form and the prescription type determines the blister packability. (DOCX) [file pdig.0001023.s001.docx]

# **Supporting information**

**On the road to vision zero: How Unit-Dose** **Dispensing Systems and health-IT are transforming clinical practices**

*Short title: Optimizing Unit-Dose with real-time dashboard insights*

*Saskia Herrmann, Natalie Bräuer, Tobias Zimmermann, Thomas Steiner, Dominic Fenske and Jana Gerstmeier*

**S1 Data:** **Original dataset for statistical analysis used in Fig 2B**

The pharmaceutical form is shown according to prescription type with the number of prescribed and documented doses. The dosage form determines the respective main group of the pharmaceutical form, and the combination of the pharmaceutical form and the prescription type determines the blister packability.

| pharmaceutical form | prescription type | prescribed doses | documented doses | pharmaceutical form main group | (non-) blisterable |
| --- | --- | --- | --- | --- | --- |
|  |  | 311 | 271 | other | non-blisterable |
|  | over the counter | 1 | 0 | other | non-blisterable |
| ampoule |  | 14 | 14 | liquid | non-blisterable |
| ampoule | narcotic drug | 470 | 440 | liquid | non-blisterable |
| ampoule |  | 270 | 268 | liquid | non-blisterable |
| ampoule | over the counter | 1.732 | 1.600 | liquid | non-blisterable |
| ampoule | over the counter | 1.954 | 1.824 | liquid | non-blisterable |
| ampoule | prescription-only medicines | 29.276 | 24.160 | liquid | non-blisterable |
| essential oil |  | 3 | 0 | liquid | non-blisterable |
| eyebath |  | 27 | 27 | liquid | non-blisterable |
| eye gel |  | 12 | 0 | semisolid | non-blisterable |
| eye gel | over the counter | 134 | 116 | semisolid | non-blisterable |
| eye gel | prescription-only medicines | 1.952 | 1.948 | semisolid | non-blisterable |
| eye ointment |  | 3 | 1 | semisolid | non-blisterable |
| eye ointment | over the counter | 206 | 119 | semisolid | non-blisterable |
| eye ointment | prescription-only medicines | 2.518 | 2.237 | semisolid | non-blisterable |
| eye drops |  | 16 | 14 | liquid | non-blisterable |
| eye drops |  | 585 | 392 | liquid | non-blisterable |
| eye drops | over the counter | 181 | 101 | liquid | non-blisterable |
| eye drops | over the counter | 10.036 | 9.074 | liquid | non-blisterable |
| eye drops | prescription-only medicines | 28.168 | 24.527 | liquid | non-blisterable |
| bath | over the counter | 21 | 18 | liquid | non-blisterable |
| bag |  | 1 | 1 | solid | non-blisterable |
| bag |  | 1.213 | 1.144 | solid | non-blisterable |
| bag | over the counter | 2 | 2 | solid | non-blisterable |
| bag | prescription-only medicines | 70 | 60 | solid | non-blisterable |
| bag with delayed-release film-coated tablets | prescription-only medicines | 9 | 6 | solid | blisterable |
| effervescent tablets |  | 207 | 192 | solid | non-blisterable |
| effervescent tablets | over the counter | 15 | 13 | solid | non-blisterable |
| effervescent tablets | over the counter | 6.895 | 6.747 | solid | non-blisterable |
| effervescent tablets | prescription-only medicines | 15.556 | 15.309 | solid | non-blisterable |
| ointment |  | 4.343 | 3.507 | semisolid | non-blisterable |
| ointment |  | 595 | 377 | semisolid | non-blisterable |
| ointment | over the counter | 5.544 | 5.066 | semisolid | non-blisterable |
| ointment | over the counter | 5 | 2 | semisolid | non-blisterable |
| ointment | prescription-only medicines | 2.733 | 2.270 | semisolid | non-blisterable |
| depot-injection suspension | prescription-only medicines | 38 | 31 | liquid | non-blisterable |
| digital health application |  | 238 | 98 | other | non-blisterable |
| dosing aerosol |  | 58 | 56 | inhalant | non-blisterable |
| dosing aerosol | prescription-only medicines | 60.781 | 53.982 | inhalant | non-blisterable |
| metering spray | over the counter | 21 | 19 | inhalant | non-blisterable |
| metering spray | prescription-only medicines | 12 | 12 | inhalant | non-blisterable |
| dragees |  | 86 | 48 | solid | blisterable |
| dragees | prescription-only medicines | 3 | 3 | solid | blisterable |
| dragees gastro-resistant | prescription-only medicines | 1.602 | 1.593 | solid | blisterable |
| vials | prescription-only medicines | 31.018 | 30.262 | liquid | non-blisterable |
| liniment |  | 10 | 10 | liquid | non-blisterable |
| single-dose pipettes |  | 56 | 26 | liquid | non-blisterable |
| single-dose pipettes | over the counter | 14 | 6 | liquid | non-blisterable |
| single-dose pipettes | prescription-only medicines | 389 | 323 | liquid | non-blisterable |
| emuslion | over the counter | 2 | 2 | liquid | non-blisterable |
| emuslion | over the counter | 4.022 | 1.818 | liquid | non-blisterable |
| emuslion | prescription-only medicines | 178 | 156 | liquid | non-blisterable |
| adult suppositories | over the counter | 1 | 0 | semisolid | non-blisterable |
| adult suppositories | prescription-only medicines | 56 | 39 | semisolid | non-blisterable |
| prefilled syringes |  | 18 | 10 | liquid | non-blisterable |
| prefilled syringes | prescription-only medicines | 16.592 | 13.557 | liquid | non-blisterable |
| prefilled syringes | blood product | 115 | 99 | liquid | non-blisterable |
| fatty ointment |  | 150 | 129 | semisolid | non-blisterable |
| film-coated tablet | prescription-only medicines | 1.934 | 1.880 | solid | blisterable |
| film-coated tablet |  | 102 | 80 | solid | blisterable |
| film-coated tablet | narcotic drug | 1.626 | 1.583 | solid | non-blisterable |
| film-coated tablet |  | 867 | 836 | solid | blisterable |
| film-coated tablet | over the counter | 140 | 82 | solid | blisterable |
| film-coated tablet | over the counter | 10.738 | 10.585 | solid | blisterable |
| film-coated tablet | prescription-only medicines | 799.601 | 788.887 | solid | blisterable |
| film-coated tablet enteric coated | prescription-only medicines | 332 | 329 | solid | blisterable |
| bottles |  | 1.102 | 1.045 | liquid | non-blisterable |
| liquid |  | 23.598 | 21.979 | liquid | non-blisterable |
| liquid | over the counter | 18 | 16 | liquid | non-blisterable |
| liquid for ingestion |  | 309 | 302 | liquid | non-blisterable |
| gas |  | 33.053 | 29.837 | inhalant | non-blisterable |
| gel |  | 57 | 52 | semisolid | non-blisterable |
| gel | over the counter | 7.039 | 6.636 | semisolid | non-blisterable |
| gel | over the counter | 49.294 | 46.895 | semisolid | non-blisterable |
| gel | prescription-only medicines | 1.323 | 1.080 | semisolid | non-blisterable |
| globuli | over the counter | 8 | 8 | solid | non-blisterable |
| granules |  | 75 | 59 | solid | non-blisterable |
| granules | over the counter | 68 | 57 | solid | non-blisterable |
| granules | over the counter | 123 | 114 | solid | non-blisterable |
| granules | prescription-only medicines | 488 | 465 | solid | non-blisterable |
| granules for the preparation of an oral suspension |  | 3 | 2 | solid | non-blisterable |
| granules for the preparation of an oral suspension | over the counter | 2.262 | 2.205 | solid | non-blisterable |
| granules for the preparation of an oral suspension | prescription-only medicines | 16 | 14 | solid | non-blisterable |
| hard capsules | narcotic drug | 16.795 | 16.688 | solid | non-blisterable |
| hard capsules | over the counter | 2.124 | 2.076 | solid | blisterable |
| hard capsules | over the counter | 12.689 | 12.552 | solid | blisterable |
| hard capsules | prescription-only medicines | 103.969 | 102.873 | solid | blisterable |
| hard capsules | T-prescription | 98 | 95 | solid | non-blisterable |
| hard capsules with enteric coated pellets | over the counter | 5.431 | 5.355 | solid | blisterable |
| hard capsules with enteric coated pellets | over the counter | 34.412 | 34.071 | solid | blisterable |
| hard capsules with powder for inhalation | prescription-only medicines | 10.708 | 9.424 | inhalant | non-blisterable |
| hard capsules with modified release of the active ingredient | narcotic drug | 871 | 863 | solid | non-blisterable |
| hard capsules with modified release of the active ingredient | prescription-only medicines | 27.853 | 27.632 | solid | blisterable |
| implant | prescription-only medicines | 1 | 1 | other | non-blisterable |
| infusion ampoules | prescription-only medicines | 2 | 0 | liquid | non-blisterable |
| infusion bag | over the counter | 367 | 341 | liquid | non-blisterable |
| infusion bag | prescription-only medicines | 132 | 122 | liquid | non-blisterable |
| infusion bottles | over the counter | 2 | 2 | liquid | non-blisterable |
| infusion bottles | over the counter | 10 | 10 | liquid | non-blisterable |
| infusion bottles | prescription-only medicines | 408 | 404 | liquid | non-blisterable |
| infusion solution |  | 9 | 9 | liquid | non-blisterable |
| infusion solution | over the counter | 67.937 | 5.836 | liquid | non-blisterable |
| infusion solution | over the counter | 37.216 | 29.678 | liquid | non-blisterable |
| infusion solution | prescription-only medicines | 28.320 | 22.594 | liquid | non-blisterable |
| infusion solution | blood product | 1.551 | 1.451 | liquid | non-blisterable |
| infusion solution concentrate | over the counter | 360 | 259 | liquid | non-blisterable |
| infusion solution concentrate | prescription-only medicines | 5.598 | 1.650 | liquid | non-blisterable |
| inhalant |  | 538 | 480 | inhalant | non-blisterable |
| inhalant | prescription-only medicines | 16 | 10 | inhalant | non-blisterable |
| inhalation ampoules |  | 24.107 | 22.885 | inhalant | non-blisterable |
| inhalation ampoules | prescription-only medicines | 144 | 128 | inhalant | non-blisterable |
| inhalation capsules | prescription-only medicines | 92 | 80 | inhalant | non-blisterable |
| inhalation solution |  | 1 | 1 | inhalant | non-blisterable |
| inhalation solution |  | 63 | 45 | inhalant | non-blisterable |
| inhalation solution | prescription-only medicines | 67.600 | 60.785 | inhalant | non-blisterable |
| inhalation powder |  | 60 | 28 | inhalant | non-blisterable |
| inhalation powder | prescription-only medicines | 3.114 | 2.260 | inhalant | non-blisterable |
| injection/infusion solution | narcotic drug | 370 | 29 | liquid | non-blisterable |
| injection/infusion solution | over the counter | 5.740 | 272 | liquid | non-blisterable |
| injection/infusion solution | prescription-only medicines | 1.937 | 1.625 | liquid | non-blisterable |
| injection bottles |  | 4 | 0 | liquid | non-blisterable |
| injection bottles | prescription-only medicines | 1.979 | 1.529 | liquid | non-blisterable |
| injection solution |  | 1.684 | 1.234 | liquid | non-blisterable |
| injection solution | narcotic drug | 6.792 | 2.861 | liquid | non-blisterable |
| injection solution | over the counter | 15 | 15 | liquid | non-blisterable |
| injection solution | over the counter | 23.475 | 17.568 | liquid | non-blisterable |
| injection solution | prescription-only medicines | 129.995 | 103.839 | liquid | non-blisterable |
| injection solution | prescription-only medicines | 131 | 116 | liquid | non-blisterable |
| injection solution | blood product | 135 | 119 | liquid | non-blisterable |
| solution for injection in a pre-filled pen | prescription-only medicines | 24.022 | 19.474 | liquid | non-blisterable |
| solution for injection in a pre-filled pen | prescription-only medicines | 12 | 12 | liquid | non-blisterable |
| solution for injection in a prefilled syringe | prescription-only medicines | 63.480 | 62.076 | liquid | non-blisterable |
| injection suspension | prescription-only medicines | 3.425 | 2.445 | liquid | non-blisterable |
| capsules |  | 76 | 62 | solid | blisterable |
| capsules | narcotic drug | 250 | 236 | solid | non-blisterable |
| capsules |  | 2.076 | 1.887 | solid | blisterable |
| capsules | over the counter | 35 | 35 | solid | blisterable |
| capsules | over the counter | 64 | 38 | solid | blisterable |
| capsules | prescription-only medicines | 1.186 | 1.091 | solid | blisterable |
| capsules | T-prescription | 44 | 42 | solid | non-blisterable |
| capsules enteric coated |  | 700 | 694 | solid | blisterable |
| capsules enteric coated | over the counter | 2.311 | 2.274 | solid | blisterable |
| capsules enteric coated | over the counter | 1.725 | 1.682 | solid | blisterable |
| capsules enteric coated | prescription-only medicines | 354 | 325 | solid | blisterable |
| chewable tablets |  | 36 | 28 | solid | non-blisterable |
| chewable tablets |  | 70 | 68 | solid | non-blisterable |
| chewable tablets | over the counter | 10.517 | 10.317 | solid | non-blisterable |
| chewable tablets | over the counter | 71 | 62 | solid | non-blisterable |
| chewable tablets | prescription-only medicines | 2.300 | 2.236 | solid | non-blisterable |
| cone |  | 1 | 0 | other | non-blisterable |
| pediatric suppositories | over the counter | 99 | 71 | semisolid | non-blisterable |
| infant suppositories | over the counter | 2 | 1 | semisolid | non-blisterable |
| enema |  | 18 | 17 | liquid | non-blisterable |
| enema |  | 218 | 201 | liquid | non-blisterable |
| enema | over the counter | 6 | 5 | liquid | non-blisterable |
| enema | prescription-only medicines | 1 | 1 | liquid | non-blisterable |
| combination pack | over the counter | 25 | 23 | other | non-blisterable |
| combination pack | prescription-only medicines | 133 | 118 | other | non-blisterable |
| concentrate | prescription-only medicines | 62 | 62 | liquid | non-blisterable |
| concentrate for the preparation of a solution for injection or infusion | over the counter | 93 | 8 | liquid | non-blisterable |
| concentrate for the preparation of a solution for injection or infusion | prescription-only medicines | 345 | 204 | liquid | non-blisterable |
| solution |  | 9.265 | 8.574 | liquid | non-blisterable |
| solution |  | 76.382 | 73.231 | liquid | non-blisterable |
| solution | over the counter | 2.094 | 2.008 | liquid | non-blisterable |
| solution | over the counter | 752 | 658 | liquid | non-blisterable |
| solution | prescription-only medicines | 1.284 | 1.018 | liquid | non-blisterable |
| solution for a nebulizer |  | 52 | 42 | inhalant | non-blisterable |
| solution for a nebulizer | over the counter | 1.498 | 1.388 | inhalant | non-blisterable |
| solution for a nebulizer | prescription-only medicines | 4.987 | 4.386 | inhalant | non-blisterable |
| oral solution | narcotic drug | 799 | 758 | liquid | non-blisterable |
| oral solution |  | 11 | 4 | liquid | non-blisterable |
| oral solution | over the counter | 30.170 | 29.491 | liquid | non-blisterable |
| oral solution | over the counter | 245 | 226 | liquid | non-blisterable |
| oral solution | prescription-only medicines | 14.514 | 13.862 | liquid | non-blisterable |
| solution for injection, infusion and inhalation | prescription-only medicines | 8 | 7 | liquid | non-blisterable |
| lotion |  | 524 | 513 | semisolid | non-blisterable |
| lotion | over the counter | 72 | 72 | semisolid | non-blisterable |
| lozenges |  | 2 | 2 | solid | non-blisterable |
| lozenges |  | 14 | 14 | solid | non-blisterable |
| lozenges | over the counter | 1.701 | 1.586 | solid | non-blisterable |
| lozenges | over the counter | 50 | 46 | solid | non-blisterable |
| lozenges | prescription-only medicines | 4.844 | 4.730 | solid | non-blisterable |
| enteric-coated hard capsules | over the counter | 86 | 73 | solid | blisterable |
| enteric-coated hard capsules | prescription-only medicines | 5.288 | 5.210 | solid | blisterable |
| enteric-coated soft capsules | over the counter | 4 | 1 | solid | blisterable |
| enteric-coated granules | prescription-only medicines | 22 | 18 | solid | non-blisterable |
| enteric-coated sustained-release granules | prescription-only medicines | 3.082 | 3.036 | solid | non-blisterable |
| nasal spray | prescription-only medicines | 68 | 54 | liquid | non-blisterable |
| nasal gel |  | 782 | 650 | semisolid | non-blisterable |
| nasal ointment | prescription-only medicines | 179 | 127 | semisolid | non-blisterable |
| nasal spray |  | 152 | 140 | liquid | non-blisterable |
| nasal spray | over the counter | 508 | 422 | liquid | non-blisterable |
| nasal spray | prescription-only medicines | 1.416 | 1.263 | liquid | non-blisterable |
| nasal drops | over the counter | 1.800 | 1.646 | liquid | non-blisterable |
| ocusert | prescription-only medicines | 12 | 3 | other | non-blisterable |
| ear drops |  | 4 | 2 | liquid | non-blisterable |
| ear drops | prescription-only medicines | 6 | 2 | liquid | non-blisterable |
| oil |  | 56 | 56 | liquid | non-blisterable |
| oil | over the counter | 18 | 17 | liquid | non-blisterable |
| ovula | prescription-only medicines | 48 | 44 | semisolid | non-blisterable |
| paste |  | 44 | 40 | semisolid | non-blisterable |
| paste | over the counter | 3.503 | 2.974 | semisolid | non-blisterable |
| paste | prescription-only medicines | 791 | 684 | semisolid | non-blisterable |
| pellets |  | 4 | 0 | solid | non-blisterable |
| plaster | prescription-only medicines | 73 | 44 | other | non-blisterable |
| plaster transdermal | narcotic drug | 2.724 | 2.527 | solid | non-blisterable |
| plaster transdermal | over the counter | 486 | 365 | other | non-blisterable |
| plaster transdermal | prescription-only medicines | 1.650 | 1.556 | other | non-blisterable |
| powder | over the counter | 24 | 8 | solid | non-blisterable |
| Powder and solvent for the preparation of a solution for intravesical use | prescription-only medicines | 1 | 0 | liquid | non-blisterable |
| powder |  | 75 | 59 | solid | non-blisterable |
| powder |  | 12.110 | 11.714 | solid | non-blisterable |
| powder | over the counter | 57 | 52 | solid | non-blisterable |
| powder | over the counter | 3.683 | 3.632 | solid | non-blisterable |
| powder | prescription-only medicines | 2.561 | 2.452 | solid | non-blisterable |
| Powder for a concentrate for the preparation of a solution for infusion | prescription-only medicines | 760 | 638 | liquid | non-blisterable |
| Powder for a concentrate for the preparation of a solution for infusion | prescription-only medicines | 2.812 | 2.720 | liquid | non-blisterable |
| Powders and solvent for injections and infusions | prescription-only medicines | 851 | 612 | liquid | non-blisterable |
| Powders and solvent for injections and infusions | blood product | 180 | 176 | liquid | non-blisterable |
| Powders and solvent for injections | prescription-only medicines | 3.155 | 2.421 | liquid | non-blisterable |
| Powders and solvent for injections | blood product | 340 | 337 | liquid | non-blisterable |
| Powder and solvent for the preparation of a depot injection suspension | prescription-only medicines | 7 | 5 | liquid | non-blisterable |
| Powder for the preparation of an injectable or inhalation solution | prescription-only medicines | 8 | 6 | liquid | non-blisterable |
| Powder for the preparation of a solution for infusion | prescription-only medicines | 61.235 | 32.667 | liquid | non-blisterable |
| Powder for the preparation of an injection or infusion solution | prescription-only medicines | 55.712 | 30.566 | liquid | non-blisterable |
| Powder for the preparation of an injection or infusion solution | blood product | 22 | 18 | liquid | non-blisterable |
| Powder for the preparation of a solution for injection | prescription-only medicines | 18.511 | 11.826 | liquid | non-blisterable |
| Powder for the preparation of an oral solution |  | 532 | 503 | liquid | non-blisterable |
| Powder for the preparation of an oral solution |  | 7 | 7 | liquid | non-blisterable |
| Powder for the preparation of an oral solution | over the counter | 185 | 160 | liquid | non-blisterable |
| Powder for the preparation of an oral solution | over the counter | 8 | 7 | liquid | non-blisterable |
| Powder for the preparation of an oral solution | prescription-only medicines | 68.365 | 66.857 | liquid | non-blisterable |
| Powder for the preparation of an oral suspension | prescription-only medicines | 3.800 | 3.638 | liquid | non-blisterable |
| Powder for the preparation of an infusion solution concentrate | over the counter | 2.808 | 639 | liquid | non-blisterable |
| Powder for the preparation of an infusion solution concentrate | prescription-only medicines | 140 | 137 | liquid | non-blisterable |
| Pumping solution | over the counter | 7 | 6 | liquid | non-blisterable |
| Pumping solution | prescription-only medicines | 2 | 2 | liquid | non-blisterable |
| rectal capsules | prescription-only medicines | 1 | 1 | solid | blisterable |
| rectal foam | prescription-only medicines | 127 | 106 | semisolid | non-blisterable |
| rectal suspension | prescription-only medicines | 288 | 270 | liquid | non-blisterable |
| ratrd granules | prescription-only medicines | 8 | 7 | solid | non-blisterable |
| retard capsules |  | 18 | 6 | solid | blisterable |
| retard capsules | narcotic drug | 50.715 | 49.491 | solid | non-blisterable |
| retard capsules |  | 448 | 433 | solid | blisterable |
| retard capsules | over the counter | 10 | 6 | solid | blisterable |
| retard capsules | prescription-only medicines | 24.559 | 24.226 | solid | blisterable |
| retard tablets |  | 22 | 20 | solid | blisterable |
| retard tablets | narcotic drug | 44.896 | 43.431 | solid | non-blisterable |
| retard tablets | over the counter | 68 | 47 | solid | blisterable |
| retard tablets | prescription-only medicines | 180.668 | 178.899 | solid | blisterable |
| juice |  | 6 | 0 | liquid | non-blisterable |
| juice | over the counter | 2 | 1 | liquid | non-blisterable |
| juice | prescription-only medicines | 363 | 357 | liquid | non-blisterable |
| ointment |  | 218 | 197 | semisolid | non-blisterable |
| ointment |  | 238 | 214 | semisolid | non-blisterable |
| ointment | over the counter | 952 | 761 | semisolid | non-blisterable |
| ointment | prescription-only medicines | 5.019 | 4.095 | semisolid | non-blisterable |
| ointment for use in the oral cavity | prescription-only medicines | 4 | 2 | semisolid | non-blisterable |
| foam | prescription-only medicines | 1 | 0 | semisolid | non-blisterable |
| lozenge | over the counter | 4 | 2 | solid | non-blisterable |
| lozenge | prescription-only medicines | 5.632 | 5.527 | solid | non-blisterable |
| sponges |  | 30 | 28 | other | non-blisterable |
| syrup |  | 10 | 9 | liquid | non-blisterable |
| syrup |  | 9 | 7 | liquid | non-blisterable |
| syrup | over the counter | 3 | 2 | liquid | non-blisterable |
| syrup | over the counter | 16.312 | 15.771 | liquid | non-blisterable |
| syrup | prescription-only medicines | 95 | 85 | liquid | non-blisterable |
| spray | narcotic drug | 401 | 329 | liquid | non-blisterable |
| spray |  | 3.730 | 3.306 | liquid | non-blisterable |
| spray | over the counter | 693 | 633 | liquid | non-blisterable |
| spray | over the counter | 34 | 26 | liquid | non-blisterable |
| spray | prescription-only medicines | 1.225 | 987 | liquid | non-blisterable |
| rinsing solution |  | 907 | 814 | liquid | non-blisterable |
| rinsing solution | prescription-only medicines | 28 | 20 | liquid | non-blisterable |
| sublingual tablets | narcotic drug | 171 | 154 | solid | non-blisterable |
| sublingual tablets | prescription-only medicines | 50 | 37 | solid | non-blisterable |
| substance |  | 28 | 12 | other | non-blisterable |
| suppositories |  | 3 | 2 | semisolid | non-blisterable |
| suppositories | over the counter | 77 | 66 | semisolid | non-blisterable |
| suppositories | over the counter | 1.253 | 1.158 | semisolid | non-blisterable |
| suppositories | prescription-only medicines | 657 | 561 | semisolid | non-blisterable |
| suspension |  | 271 | 260 | liquid | non-blisterable |
| suspension | over the counter | 193 | 182 | liquid | non-blisterable |
| suspension | over the counter | 109 | 102 | liquid | non-blisterable |
| suspension | prescription-only medicines | 293 | 260 | liquid | non-blisterable |
| suspension for a nebulizer | prescription-only medicines | 3.688 | 3.063 | inhalant | non-blisterable |
| oral suspension | over the counter | 3.921 | 3.671 | liquid | non-blisterable |
| oral suspension | over the counter | 30 | 30 | liquid | non-blisterable |
| oral suspension | prescription-only medicines | 548 | 523 | liquid | non-blisterable |
| tablet | prescription-only medicines | 279 | 276 | solid | blisterable |
| tablet with modified release | prescription-only medicines | 3.324 | 3.278 | solid | blisterable |
| tablet for the preparation of an oral suspension | prescription-only medicines | 7.641 | 7.532 | solid | blisterable |
| tablets |  | 204 | 192 | solid | blisterable |
| tablets | narcotic drug | 394 | 391 | solid | non-blisterable |
| tablets |  | 4.950 | 4.817 | solid | blisterable |
| tablets | over the counter | 50.526 | 50.244 | solid | blisterable |
| tablets | over the counter | 214.446 | 212.567 | solid | blisterable |
| tablets | prescription-only medicines | 1.193.602 | 1.178.905 | solid | blisterable |
| tablets in calender pack | prescription-only medicines | 5 | 5 | solid | blisterable |
| tablets enteric-coated |  | 4 | 2 | solid | blisterable |
| tablets enteric-coated |  | 28 | 28 | solid | blisterable |
| tablets enteric-coated | over the counter | 7 | 7 | solid | blisterable |
| tablets enteric-coated | over the counter | 25.447 | 25.259 | solid | blisterable |
| tablets enteric-coated | prescription-only medicines | 236.548 | 234.153 | solid | blisterable |
| platelet | prescription-only medicines | 7.402 | 7.211 | solid | non-blisterable |
| tea |  | 13 | 6 | other | non-blisterable |
| tea | over the counter | 6 | 6 | other | non-blisterable |
| test strip |  | 22 | 4 | other | non-blisterable |
| transdermal spray | prescription-only medicines | 11 | 8 | liquid | non-blisterable |
| drinking ampoules | prescription-only medicines | 6 | 2 | liquid | non-blisterable |
| dry substance without solvent | prescription-only medicines | 253 | 210 | liquid | non-blisterable |
| dried substance | prescription-only medicines | 24 | 23 | liquid | non-blisterable |
| dry substance with solvent | prescription-only medicines | 95 | 95 | liquid | non-blisterable |
| dry substance with solvent | blood product | 6 | 6 | liquid | non-blisterable |
| dry substance without solvent | over the counter | 8 | 0 | liquid | non-blisterable |
| dry substance without solvent | prescription-only medicines | 6.482 | 4.112 | liquid | non-blisterable |
| drops |  | 102 | 60 | liquid | non-blisterable |
| drops | over the counter | 82 | 58 | liquid | non-blisterable |
| drops | over the counter | 3 | 1 | liquid | non-blisterable |
| drops | prescription-only medicines | 4.785 | 4.480 | liquid | non-blisterable |
| oral drops | narcotic drug | 954 | 825 | liquid | non-blisterable |
| oral drops |  | 4 | 4 | liquid | non-blisterable |
| oral drops | over the counter | 2.847 | 2.779 | liquid | non-blisterable |
| oral drops | over the counter | 2.263 | 2.123 | liquid | non-blisterable |
| oral drops | prescription-only medicines | 116.946 | 112.361 | liquid | non-blisterable |
| coated tablets |  | 6.241 | 6.110 | solid | blisterable |
| coated tablets | over the counter | 2.104 | 2.077 | solid | blisterable |
| coated tablets | over the counter | 13.742 | 13.613 | solid | blisterable |
| coated tablets | prescription-only medicines | 16.006 | 15.711 | solid | blisterable |
| vaginal cream | over the counter | 35 | 31 | semisolid | non-blisterable |
| vaginal cream | prescription-only medicines | 96 | 77 | semisolid | non-blisterable |
| vaginal solution | over the counter | 22 | 22 | liquid | non-blisterable |
| vagonal suppositories | prescription-only medicines | 33 | 32 | semisolid | non-blisterable |
| vaginal tablets | over the counter | 67 | 63 | solid | non-blisterable |
| vaginal tablets | prescription-only medicines | 243 | 217 | solid | non-blisterable |
| bandage |  | 2 | 2 | other | non-blisterable |
| soft capsules |  | 202 | 202 | solid | blisterable |
| soft capsules | over the counter | 6 | 4 | solid | blisterable |
| soft capsules | prescription-only medicines | 18.787 | 18.210 | solid | blisterable |
| gauze | over the counter | 20 | 9 | other | non-blisterable |
| cylinder ampoules | prescription-only medicines | 107.012 | 74.517 | liquid | non-blisterable |
