# Supplementary material for: On the road to vision zero: How unit-dose dispensing systems and health-IT are transforming clinical practices
Source: PLOS Digit Health. 2025 Oct 17;4(10):e0001023. doi: 10.1371/journal.pdig.0001023 (PMC12533864; doi:10.1371/journal.pdig.0001023)
Supplement: S1 Table — (DOCX) [file pdig.0001023.s002.docx]

# **Supporting information**

**On the road to vision zero: How Unit-Dose** **Dispensing Systems and health-IT are transforming clinical practices**

*Short title: Optimizing Unit-Dose with real-time dashboard insights*

*Saskia Herrmann, Natalie Bräuer, Tobias Zimmermann, Thomas Steiner, Dominic Fenske and Jana Gerstmeier*

**S1 Table: Glossary of terms**

| **term** | **definition** | **example** |
| --- | --- | --- |
| prescription | medication order in the Electronic Medical System (EMS), including dosing intervals. Any changes (e.g., dose adjustments) create a new prescription | pantoprazole 1-0-1 (morning and evening doses) |
| prescribed doses | total number of doses derived from the prescription duration and frequency | pantoprazole 1-0-1 for 10 days = 20 prescribed doses |
| documented doses | number of doses that have been recorded as documentes in the EMS after administered | pantoprazole 1-0-1 for 10 days, but for example no documentation on day 3 results in 18 documented doses |
| documentation rate | percentage of documented doses relative to prescribed doses | 18 documented out of 20 prescribed doses results in 90% documentation rate |
| cut-off time | deadline for prescription submission in the EMS to ensure inclusion in the daily production of patient-specific blister packs (UDDS) | EMS entry after 15 o´clock: medication will not be blister-packed that day, but 24 hours later |
| special order | drugs, that are not listed in the standard hospital formulary and therefore not supplied by UDDS; separate ordering by the ward is required | General practitioner prescribes Roxadustat, which is not part of the hospital formulary, so the nurs/physician on ward requests the speial order in pharmacy |
