# Supplementary material for: On the road to vision zero: How unit-dose dispensing systems and health-IT are transforming clinical practices
Source: PLOS Digit Health. 2025 Oct 17;4(10):e0001023. doi: 10.1371/journal.pdig.0001023 (PMC12533864; doi:10.1371/journal.pdig.0001023)
Supplement: S2 Table — Solid forms eligible for unit-dose are ranked in descending order by absolute prescription frequency. Drugs with modified release of the active ingredient are categorised within the superior pharmaceutical form (e.g., enteric-resistant capsules are classified as capsules). (DOCX) [file pdig.0001023.s003.docx]

# **Supporting information**

**On the road to vision zero: How Unit-Dose** **Dispensing Systems and health-IT are transforming clinical practices**

*Short title: Optimizing Unit-Dose with real-time dashboard insights*

*Saskia Herrmann, Natalie Bräuer, Tobias Zimmermann, Thomas Steiner, Dominic Fenske and Jana Gerstmeier*

**S2 Table: Prescribed doses by subordinate solid pharmaceutical form****.** Solid forms eligible for unit-dose are ranked in descending order by absolute prescription frequency. Drugs with modified release of the active ingredient are categorised within the superior pharmaceutical form (e.g. enteric-resistant capsules are classified as capsules).

| pharmaceutical form | prescribed doses | percentage |
| --- | --- | --- |
| tablets | 1,917,769 | 64% |
| film coated tablets | 851,816 | 28% |
| hard capsules | 225,415 | 7% |
| soft capsules | 18,999 | 1% |
| dragees | 1,691 | < 1% |
