# Supplementary material for: On the road to vision zero: How unit-dose dispensing systems and health-IT are transforming clinical practices
Source: PLOS Digit Health. 2025 Oct 17;4(10):e0001023. doi: 10.1371/journal.pdig.0001023 (PMC12533864; doi:10.1371/journal.pdig.0001023)
Supplement: S3 Table — Total number of wards assigned to each hospital department at HK-EF. Each row corresponds to a specific department, and the adjacent column indicates the number of wards managed by that department. Dept. I is subdivided in Ia-c. (DOCX) [file pdig.0001023.s004.docx]

# **Supporting information**

**On the road to vision zero: How Unit-Dose** **Dispensing Systems and health-IT are transforming clinical practices**

*Short title: Optimizing Unit-Dose with real-time dashboard insights*

*Saskia Herrmann, Natalie Bräuer, Tobias Zimmermann, Thomas Steiner, Dominic Fenske and Jana Gerstmeier*

**S3 Table: Wards per department****.** Total number of wards assigned to each hospital department at HK-EF. Each row corresponds to a specific department, and the adjacent column indicates the number of wards managed by that department. Dept. I is subdivided in Ia-c.

| department | [n] wards |
| --- | --- |
| department Ia | 8 |
| department Ib | 7 |
| department Ic | 3 |
| department II | 8 |
| department III | 5 |
| department IV | 4 |
| department V | 3 |
| department VI | 7 |
| department VII | 2 |
