# Supplementary material for: On the road to vision zero: How unit-dose dispensing systems and health-IT are transforming clinical practices
Source: PLOS Digit Health. 2025 Oct 17;4(10):e0001023. doi: 10.1371/journal.pdig.0001023 (PMC12533864; doi:10.1371/journal.pdig.0001023)
Supplement: S4 Table — The top 25 most frequently prescribed ATC codes at HK-EF in 2023 are presented in a heat map, ranked in descending order by absolute prescription frequency. The first row displays the absolute numbers of prescriptions, with blue indicating the highest and light green the lowest. The second row shows the corresponding 2nd level ATC codes, while the third row provides their full descriptions. (DOCX) [file pdig.0001023.s005.docx]

# **Supporting information**

**On the road to vision zero: How Unit-Dose** **Dispensing Systems and health-IT are transforming clinical practices**

*Short title: Optimizing Unit-Dose with real-time dashboard insights*

*Saskia Herrmann, Natalie Bräuer, Tobias Zimmermann, Thomas Steiner, Dominic Fenske and Jana Gerstmeier*

**S4 Table: Top 25 prescribed ATC-codes in HK-EF in 2023.** The top 25 most frequently prescribed ATC codes at HK-EF in 2023 are presented in a heat map, ranked in descending order by absolute prescription frequency. The first row displays the absolute numbers of prescriptions, with blue indicating the highest and light green the lowest. The second row shows the corresponding 2^nd^ level ATC codes, while the third row provides their full descriptions.

***max min***

|  |  |  |  |
| --- | --- | --- | --- |
| **total prescriptions** | | | |
| [n] | 2^nd^ level |  | |
| prescriptions | ATC-code |  | |
| **39,019** | B01 | antithrombotic agents | |
| **38,139** | N02 | analgesics | |
| **23,704** | C09 | agents acting on the renin-angiotensin-system | |
| **23,311** | A02 | drugs for acid related dirsorders | |
| **22,477** | B05 | blood substitutes and perfusion solution | |
| **20,536** | A10 | drugs used in diabetes | |
| **20,268** | C03 | diuretics | |
| **18,301** | C07 | beta blocking agents | |
| **16,640** | C10 | lipid modifying agents | |
| **16,160** | J01 | antibacterials for systemic use | |
| **13,522** | N05 | psycholeptics | |
| **9,323** | C08 | calcium channel blockers | |
| **8,426** | A06 | drugs for constipation | |
| **7,801** | R03 | drugs for obstructive airway disease | |
| **7,711** | A11 | vitamines | |
| **7,590** | H03 | thyroid therapy | |
| **7,264** | B03 | antianemic preparations | |
| **7,224** | H02 | corticosteroids for systemic use | |
| **6,574** | N06 | psychoanaleptics | |
| **6,474** | M01 | antiinflammatory &rheumatic products, non steroids | |
| **4,174** | R05 | cough and cold preparations | |
| **4,144** | N03 | antiepileptics | |
| **4,009** | V06 | general nutrients | |
| **3,893** | A04 | antiemetics and antinauseants | |
| **3,736** | M04 | antigout preparations | |
