# Supplementary material for: On the road to vision zero: How unit-dose dispensing systems and health-IT are transforming clinical practices
Source: PLOS Digit Health. 2025 Oct 17;4(10):e0001023. doi: 10.1371/journal.pdig.0001023 (PMC12533864; doi:10.1371/journal.pdig.0001023)
Supplement: S1 Fig — The hospital information system (HIS) transfers administrative patient data to the electronic medication system (EMS), which is used by physicians for drug prescribing. The EMS can generate clinical alerts, such as contraindication warnings or weight-adjusted dosing recommendations, but does not support prescription data analysis. ID EFIX PHARMA consolidates data from both the HIS and EMS, providing structured data fields that enables various types of evaluations based on defined criteria for dashboards. (DOCX) [file pdig.0001023.s006.docx]

# **Supporting information**

**On the road to vision zero: How Unit-Dose** **Dispensing Systems and health-IT are transforming clinical practices**

*Short title: Optimizing Unit-Dose with real-time dashboard insights*

*Saskia Herrmann, Natalie Bräuer, Tobias Zimmermann, Thomas Steiner, Dominic Fenske and Jana Gerstmeier*

**S1 Fig:**


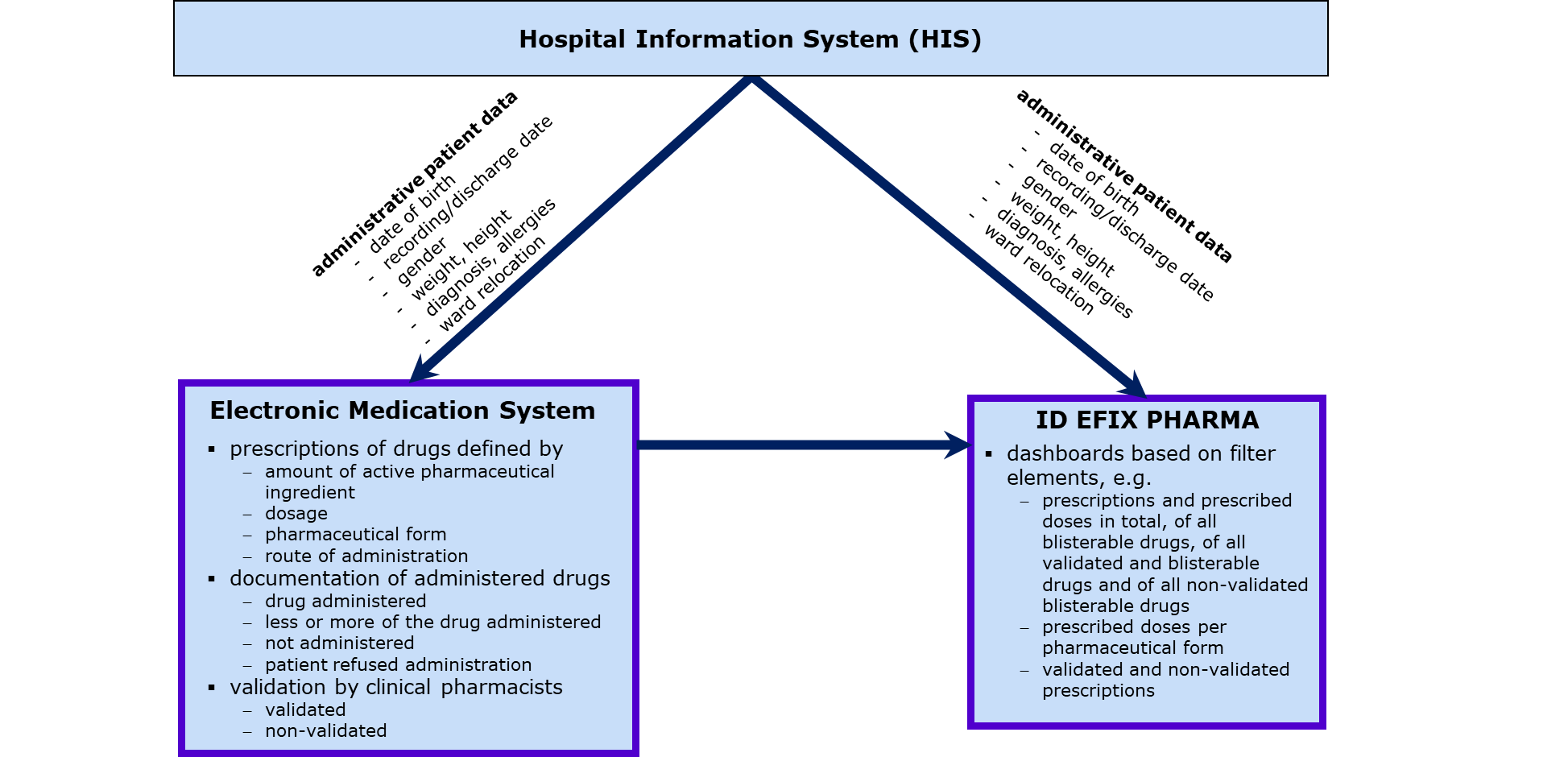


**S1 Fig:** **Schematic overview of data exchange.** The hospital information system (HIS) transfers administrative patient data to the electronic medication system (EMS), which is used by physicians for drug prescribing. The EMS can generate clinical alerts, such as contraindication warnings or weight-adjusted dosing recommendations, but does not support prescription data analysis. ID EFIX PHARMA consolidates data from both the HIS and EMS, providing structured data fields that enables various types of evaluations based on defined criteria for dashboards.
