# Supplementary material for: On the road to vision zero: How unit-dose dispensing systems and health-IT are transforming clinical practices
Source: PLOS Digit Health. 2025 Oct 17;4(10):e0001023. doi: 10.1371/journal.pdig.0001023 (PMC12533864; doi:10.1371/journal.pdig.0001023)
Supplement: S2 Fig — Filter setting I includes all prescribed drugs without limitations. Filter setting II refines the selection to prescribed and blisterable drugs. Filter setting III further narrows the focus to prescribed, blisterable, and validated drugs. Filter setting IV identifies prescribed, blisterable, but non-validated drugs. w/o = without, special order = drugs not routinely stocked. (DOCX) [file pdig.0001023.s007.docx]

# **Supporting information**

**On the road to vision zero: How Unit-Dose** **Dispensing Systems and health-IT are transforming clinical practices**

*Short title: Optimizing Unit-Dose with real-time dashboard insights*

*Saskia Herrmann, Natalie Bräuer, Tobias Zimmermann, Thomas Steiner, Dominic Fenske and Jana Gerstmeier*

**S2 Fig:**


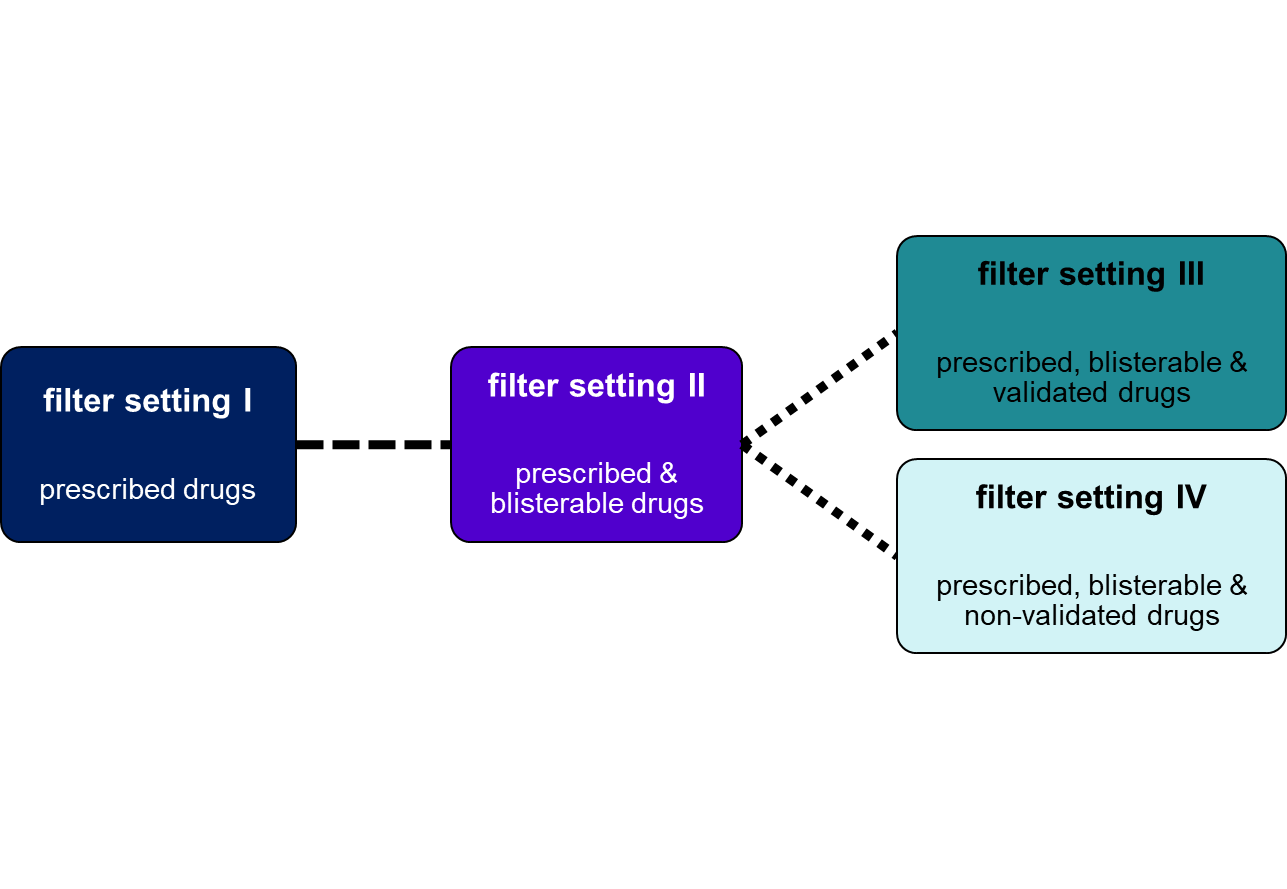

**S2 Fig:** **Graphical overview and tabular description of filter settings I - IV.** Filter setting I includes all prescribed drugs without limitations. Filter setting II refines the selection to prescribed and blisterable drugs. Filter setting III further narrows the focus to prescribed, blisterable, and validated drugs. Filter setting IV identifies prescribed, blisterable, but non-validated drugs. w/o = without, special order = drugs not routinely stocked
