# Supplementary material for: On the road to vision zero: How unit-dose dispensing systems and health-IT are transforming clinical practices
Source: PLOS Digit Health. 2025 Oct 17;4(10):e0001023. doi: 10.1371/journal.pdig.0001023 (PMC12533864; doi:10.1371/journal.pdig.0001023)
Supplement: S3 Fig — The flowchart illustrates the stepwise application of filter setting I in combination with prescription status to classify medications by pharmaceutical form. Drugs are first grouped into five categories: solid, liquid, semi-solid, inhalant, and other. Solid drugs are further divided based on blisterability and prescription type, resulting in classification as UDDS-compatible (solid UDDS) or not compatible (solid w/o UDDS). (DOCX) [file pdig.0001023.s008.docx]

# **Supporting information**

**On the road to vision zero: How Unit-Dose** **Dispensing Systems and health-IT are transforming clinical practices**

*Short title: Optimizing Unit-Dose with real-time dashboard insights*

*Saskia Herrmann, Natalie Bräuer, Tobias Zimmermann, Thomas Steiner, Dominic Fenske and Jana Gerstmeier*

**S3 Fig:**


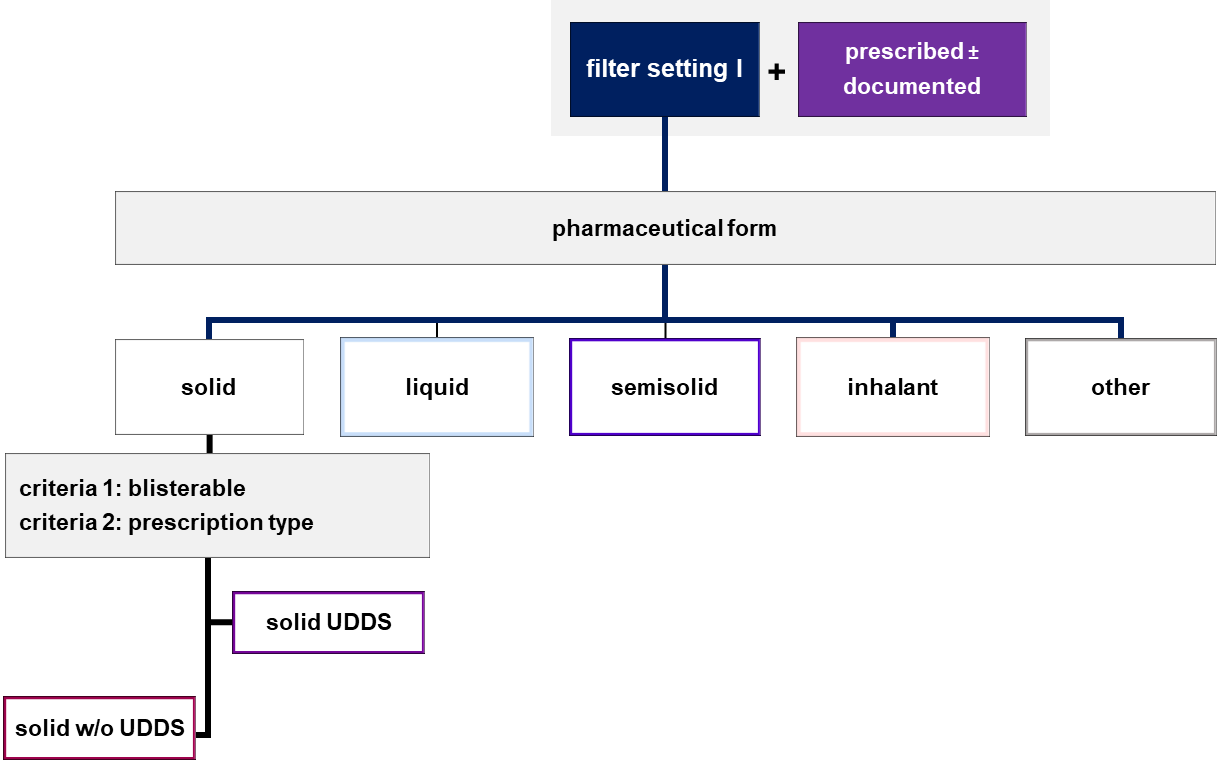


**S3 Fig: Schematic overview of filter setting applied to pharmaceutical form.** The flowchart illustrates the stepwise application of filter setting I in combination with prescription status to classify medications by pharmaceutical form. Drugs are first grouped into five categories: solid, liquid, semi-solid, inhalant, and other. Solid drugs are further divided based on blisterability and prescription type, resulting in classification as UDDS-compatible (solid UDDS) or not compatible (solid w/o UDDS).
