# Supplementary material for: On the road to vision zero: How unit-dose dispensing systems and health-IT are transforming clinical practices
Source: PLOS Digit Health. 2025 Oct 17;4(10):e0001023. doi: 10.1371/journal.pdig.0001023 (PMC12533864; doi:10.1371/journal.pdig.0001023)
Supplement: S4 Fig — (A) Overview of parameters and filter settings I–III used to extract descriptive prescription data. (B) Workflow to calculate average daily prescribed doses using filter settings I, III, and IV. (C) Approach to analyze prescription time distribution, including time clustering and weekday assignment. Steps performed in Microsoft Excel are highlighted in grey. (DOCX) [file pdig.0001023.s009.docx]

# **Supporting information**

**On the road to vision zero: How Unit-Dose** **Dispensing Systems and health-IT are transforming clinical practices**

*Short title: Optimizing Unit-Dose with real-time dashboard insights*

*Saskia Herrmann, Natalie Bräuer, Tobias Zimmermann, Thomas Steiner, Dominic Fenske and Jana Gerstmeier*

**S4 Fig:**


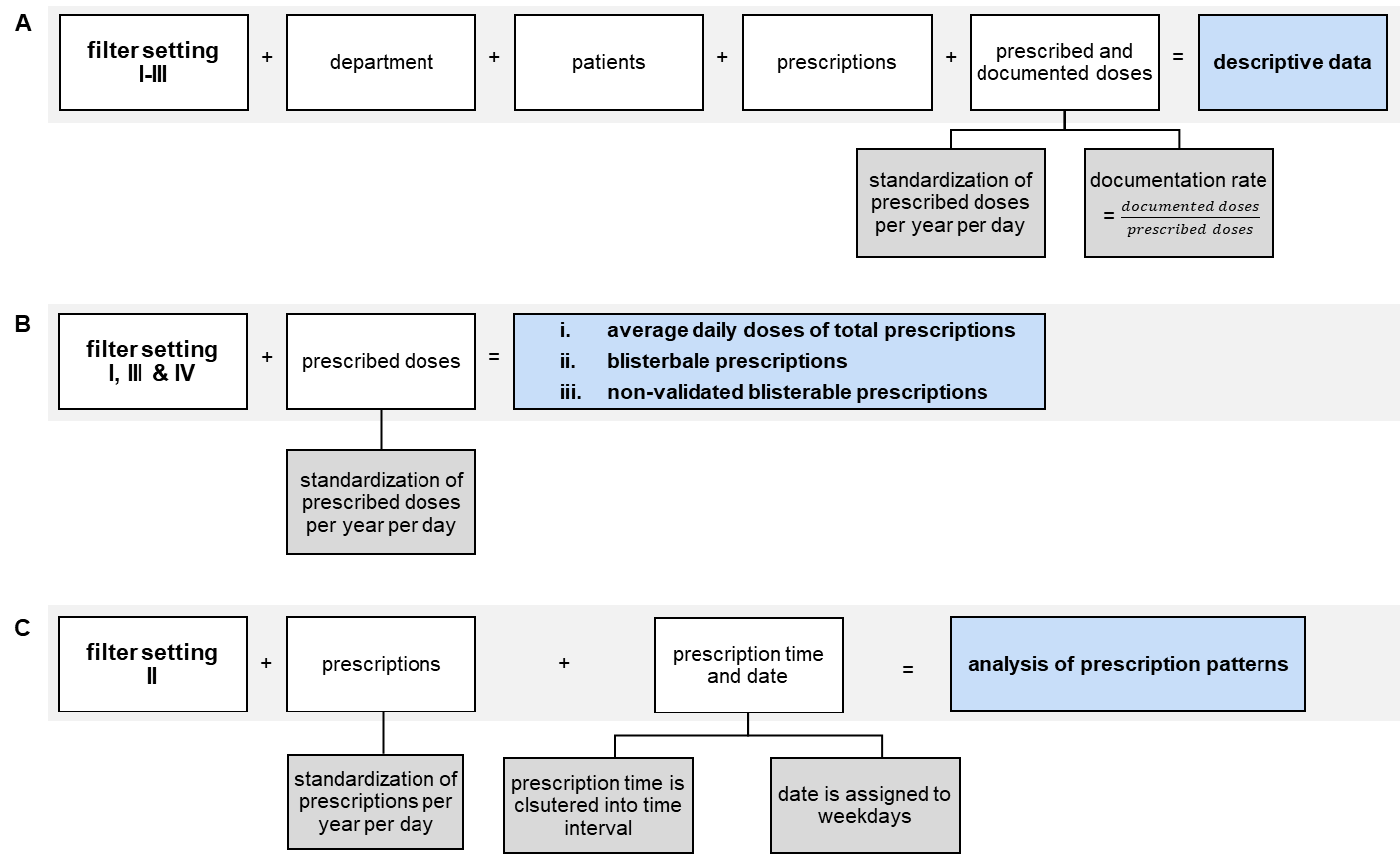


**S4 Fig: Flowcharts for the dashboard analysis of prescription timing and volume.** (A) Overview of parameters and filter settings I–III used to extract descriptive prescription data. (B) Workflow to calculate average daily prescribed doses using filter settings I, III, and IV. (C) Approach to analyze prescription time distribution, including time clustering and weekday assignment. Steps performed in Microsoft Excel are highlighted in grey.
