# Supplementary material for: On the road to vision zero: How unit-dose dispensing systems and health-IT are transforming clinical practices
Source: PLOS Digit Health. 2025 Oct 17;4(10):e0001023. doi: 10.1371/journal.pdig.0001023 (PMC12533864; doi:10.1371/journal.pdig.0001023)
Supplement: S5 Fig — Filter settings I, II and IV were used to identify and compare the 20 most frequently prescribed 2nd ATC codes (lightblue), across all prescriptions (grey), blisterable prescriptions (dark purple), and non-validated blisterable prescriptions (light purple). (DOCX) [file pdig.0001023.s010.docx]

# **Supporting information**

**On the road to vision zero: How Unit-Dose** **Dispensing Systems and health-IT are transforming clinical practices**

*Short title: Optimizing Unit-Dose with real-time dashboard insights*

*Saskia Herrmann, Natalie Bräuer, Tobias Zimmermann, Thomas Steiner, Dominic Fenske and Jana Gerstmeier*

**S5 Fig:**


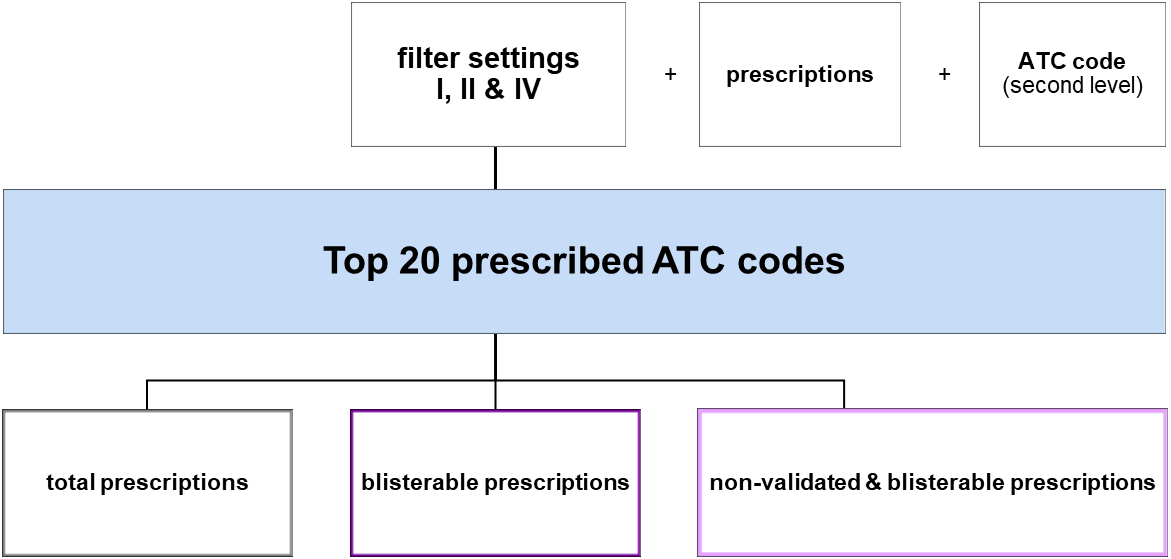


**S5 Fig: Flowchart for 2^nd^ level ATC codes analysis.** Filter settings I, II and IV were used to identify and compare the 20 most frequently prescribed 2^nd^ ATC codes (lightblue), across all prescriptions (grey), blisterable prescriptions (dark purple), and non-validated blisterable prescriptions (light purple).

**S7 Figure:**

**S7 Fig. Average prescribed doses per weekkday versus weekend.** Bar chart illustrates the mean number of blisterable prescribed doses on working days (black) and weekends (pink) for each clinical department in 2023.
dept. = department
