# Supplementary material for: On the road to vision zero: How unit-dose dispensing systems and health-IT are transforming clinical practices
Source: PLOS Digit Health. 2025 Oct 17;4(10):e0001023. doi: 10.1371/journal.pdig.0001023 (PMC12533864; doi:10.1371/journal.pdig.0001023)
Supplement: S6 Fig — The supply of a ward with UDDS is contingent upon the determination of the feasibility of blister packing the medication. In the event that the items are not available in bulk, they must be deblistered. Concurrently, clinical pharmacists evaluate patients’ medications for potential discrepancies, including overdoses or underdoses, duplicate prescriptions, and interactions. All medications that have been validated are packaged individually for each patient using UDDS and delivered to the wards after a final visual inspection for correctness of the blister packed drug. Next, the blister packs are adminsistered to the patients by the nursing staff. Moreover, the nursing staff controls the delivered UDDS for correctness with the prescription entry in the EMS. If the medication is not produced due to pharmaceutical concerns, the nursing staff consults the attending physicians. The attending physician determines whether the medication is distributed from the ward stock or whether a dose adjustment or discontinuation is necessary according to the pharmacist´s concerns. (DOCX) [file pdig.0001023.s011.docx]

# **Supporting information**

**On the road to vision zero: How Unit-Dose** **Dispensing Systems and health-IT are transforming clinical practices**

*Short title: Optimizing Unit-Dose with real-time dashboard insights*

*Saskia Herrmann, Natalie Bräuer, Tobias Zimmermann, Thomas Steiner, Dominic Fenske and Jana Gerstmeier*

**S6 Fig:**

*
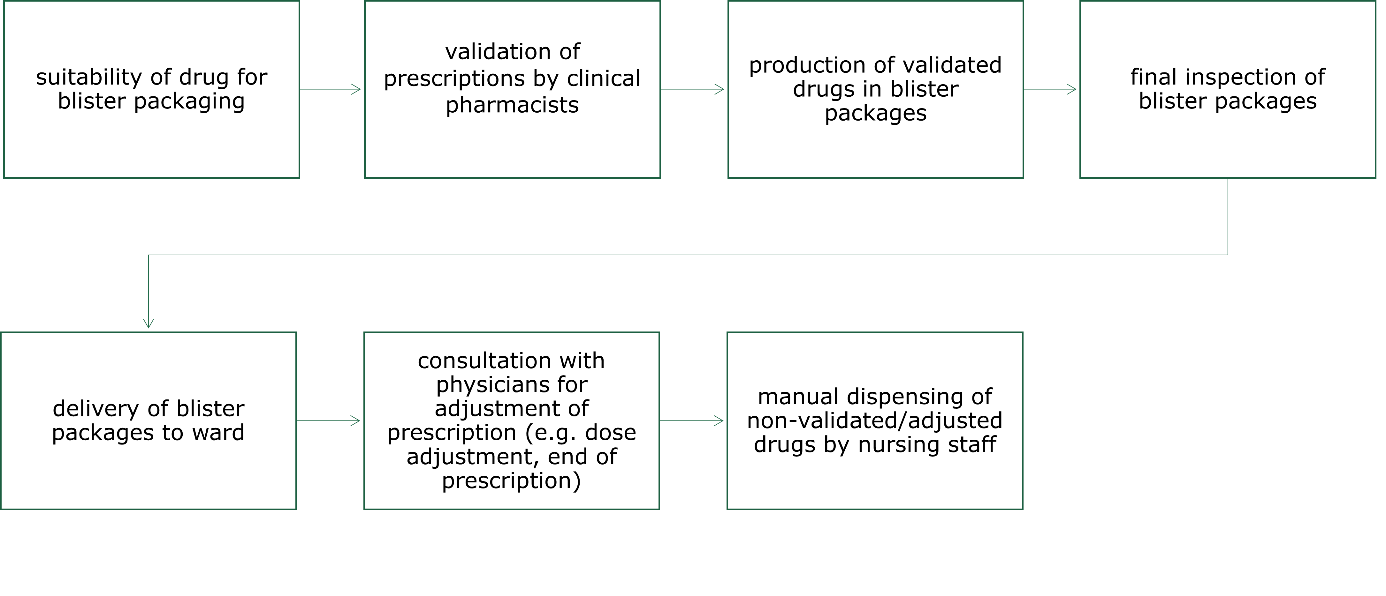
*

**S6 Fig: Workflow overview of UDDS at HK-EF.** The supply of wards with UDDS is contingent upon the determination of the feasibility of blister packing the medication. If the drugs are not available in bulk, they must be deblistered. Concurrently, clinical pharmacists evaluate patients' medications for potential discrepancies, including overdoses or underdoses, duplicate prescriptions, and interactions. All medications that have been validated are packaged individually for each patient using UDDS and delivered to the wards after a final visual inspection for correctness of the blister packed drug. Next, the blister packs are adminsistered to the patients by the nursing staff. Moreover, the nursing staff controls the delivered UDDS for correctness with the prescription entry in the EMS. If the medication is not produced due to pharmaceutical concerns, the nursing staff consults the attending physicians. The attending physician determines whether the medication is distributed from the ward stock or whether a dose adjustment or discontinuation is necessary according to the pharmacist´s concerns.
