# Supplementary material for: On the road to vision zero: How unit-dose dispensing systems and health-IT are transforming clinical practices
Source: PLOS Digit Health. 2025 Oct 17;4(10):e0001023. doi: 10.1371/journal.pdig.0001023 (PMC12533864; doi:10.1371/journal.pdig.0001023)
Supplement: S7 Fig — Bar chart illustrates the mean number of blisterable prescribed doses on working days (black) and weekends (pink) for each clinical department in 2023. dept. = department. (DOCX) [file pdig.0001023.s012.docx]

# **Supporting information**

**On the road to vision zero: How Unit-Dose** **Dispensing Systems and health-IT are transforming clinical practices**

*Short title: Optimizing Unit-Dose with real-time dashboard insights*

*Saskia Herrmann, Natalie Bräuer, Tobias Zimmermann, Thomas Steiner, Dominic Fenske and Jana Gerstmeier*

**S7 Fig:**

**S7 Fig: Average prescribed doses per weekkday versus weekend.** Bar chart illustrates the mean number of blisterable prescribed doses on working days (black) and weekends (pink) for each clinical department in 2023.
dept. = department
